# Mutations inhibiting KDM4B drive ALT activation in *ATRX*-mutated glioblastomas

**DOI:** 10.1038/s41467-021-22543-z

**Published:** 2021-05-10

**Authors:** M. Udugama, L. Hii, A. Garvie, M. Cervini, B. Vinod, F.-L. Chan, P. P. Das, J. R. Mann, P. Collas, H. P. J. Voon, L. H. Wong

**Affiliations:** 1 Department of Biochemistry and Molecular Biology, Biomedicine Discovery Institute, Monash University, Clayton, Victoria Australia; 2 Department of Anatomy and Developmental Biology, Biomedicine Discovery Institute, Monash University, Clayton, Victoria Australia; 3 Department of Molecular Medicine, Institute of Basic Medical Sciences, Faculty of Medicine, University of Oslo, Oslo, Norway; 4 Department of Immunology and Transfusion Medicine, Oslo University Hospital, Oslo, Norway

**Keywords:** Telomeres, Histone variants

## Abstract

Alternative Lengthening of Telomeres (ALT) is a telomere maintenance pathway utilised in 15% of cancers. ALT cancers are strongly associated with inactivating mutations in *ATRX*; yet loss of ATRX alone is insufficient to trigger ALT, suggesting that additional cooperating factors are involved. We identify H3.3^G34R^ and IDH1/2 mutations as two such factors in ATRX-mutated glioblastomas. Both mutations are capable of inactivating histone demethylases, and we identify KDM4B as the key demethylase inactivated in ALT. Mouse embryonic stem cells inactivated for ATRX, TP53, TERT and KDM4B (KDM4B knockout or H3.3^G34R^) show characteristic features of ALT. Conversely, KDM4B over-expression in ALT cancer cells abrogates ALT-associated features. In this work, we demonstrate that inactivation of KDM4B, through H3.3^G34R^ or IDH1/2 mutations, acts in tandem with ATRX mutations to promote ALT in glioblastomas.

## Abstract (web summary)

Alternative Lengthening of Telomeres (ALT) is a telomere maintenance pathway utilised in 15% of cancers that have been associated with mutations in ATRX. Here the authors reveal a functional role of histone demethylases KDM4B in regulating ALT activation.

## Introduction

Telomeres are stretches of repetitive DNA that cap and protect the ends of linear chromosomes. In vertebrates, telomeres are comprised of a tandemly repeated hexamer (TTAGGG) several kilobases long, which is recognised and bound by Shelterin^1,2^, a multimeric protein complex that prevents the natural ends of chromosomes from being recognised as DNA breaks. These DNA repeats buffer the genome from the ‘end-replication problem’ where incomplete replication of the lagging DNA strand leads to gradual shortening of chromosomes with each round of DNA replication. The loss of telomeric sequence with successive rounds of cell division eventually leads to critically short telomeres which cannot bind Shelterin. This results in a loss of telomere integrity and imposes a natural biological limit on cellular proliferation. Highly proliferative cells must activate a telomere maintenance mechanism. During early embryogenesis, this is facilitated by high-level expression of telomerase, a reverse transcriptase that uses an RNA template to elongate telomeres^3^. Telomerase is downregulated during late stages of development and is often only reactivated in cancers. Much like stem cells, one hallmark of cancers is the capacity for unlimited cell division. This oncogenic process must be supported by a telomere maintenance programme, either through reactivation of telomerase or activation of the Alternative Lengthening of Telomeres (ALT) pathway^4,5^.

The ALT pathway is utilised in 15% of cancers^6–9^, with high prevalence (>50%) in gliomas, pancreatic neuroendocrine tumours, osteosarcomas and soft-tissue sarcomas^7,8,10,11^. The maintenance of ALT is dependent on a host of DNA repair proteins^12,13^ sequestered with telomeres in specialised nuclear compartments known as ALT-associated PML bodies (APBs)^5,14,15^. However, the initiation and early stages of ALT remains an enigmatic process and only one protein, ATRX (α-thalassaemia/mental retardation syndrome-X-linked), has been consistently linked to ALT activation. ATRX is a chromatin remodeller that acts in complex with DAXX to deposit the histone variant H3.3 and form a heterochromatin structure at telomeres^16–22^. Inactivating mutations in ATRX are highly correlated with ALT, implicating ATRX as a suppressor of the ALT mechanism^6–9^. However, neither knockdown nor knockout of ATRX is sufficient to activate ALT^9,23^, suggesting that additional cooperating factors are necessary. To identify these factors, we searched public cancer genome databases for mutations that segregated with ATRX inactivation in glioblastoma multiforme (GBM), where ALT is particularly prevalent^6,10^. We found two frequent mutations within this cohort; one in histone H3.3 (H3.3^G34R^; glycine to arginine substitution) and one in isocitrate dehydrogenase (IDH1/2). We re-created the H3.3^G34R^ and ATRX mutations in mouse ES cells that lack telomerase (TERT) and TP53, and show in this model system that H3.3^G34R^ cooperates with ATRX inactivation to induce ALT. We further show that both H3.3^G34R^ and IDH1^R132H^ promote ALT through the inhibition of KDM4B, a histone K9/K36 demethylase. Our results demonstrate that H3.3^G34R^ and IDH1/2 are parallel mutations that promote ALT by inhibiting KDM4B.

## Results

### H3.3^G34R^ collaborates with ATRX inactivation to induce ALT

**Fig. 1:**
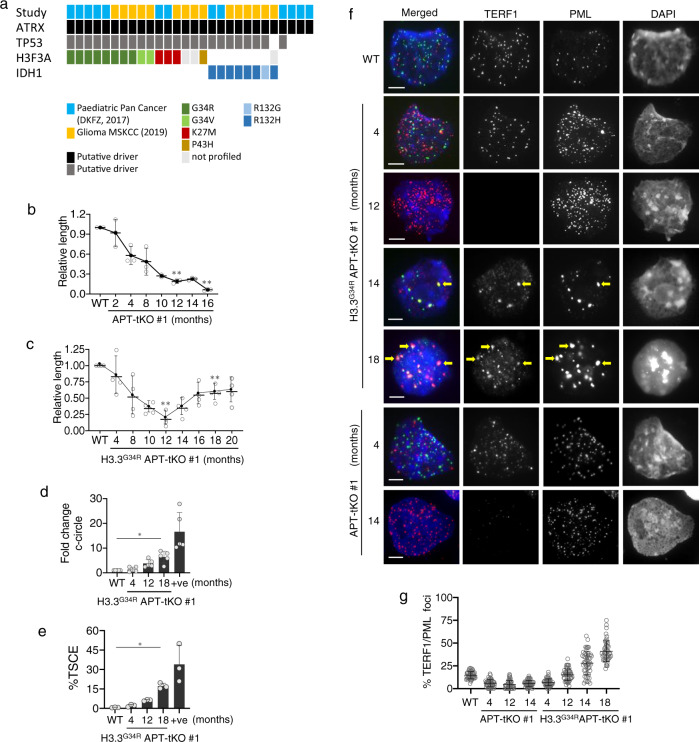
H3.3^G34R^ promotes ALT in *APT-tKO* mouse ES cells. **a** Glioblastoma tumours from patients aged 30 years and younger with ATRX, H3.3, IDH1 and TP53 mutations. **b, c** Telomere length analyses of APT-tKO #1 and H3.3^G34R^APT-tKO #1 ES cells, in relative to WT (wildtype) ES cells (*n* = 3 independent experiments). **d** Detection of telomeric C-circle in H3.3^G34R^APT-tKO cells (*n* = 5 independent experiments). **e** Detection of T-SCE in H3.3^G34R^APT-tKO cells (*n* = 3 independent experiments). Human U2OS osteosarcoma ALT cell line was included as a positive (+ve) control. **f, g** Immunostaining of APBs in APT-tKO cells and H3.3^G34R^APT-tKO, shown by co-staining of TERF1 (green; diluted at 1/500) and PML (red; diluted at 1/800) (arrows). Scale bars: 5 μm; representative images from at least *n* = 3 independent experiments. Percentages of co-localised TERF1 and PML foci in H3.3^G34R^APT-tKO#1 cells are shown in **g**. For each cell line, >1500 telomeric foci were counted (*n* = 3 independent experiments; indicated by TERF1 staining). Percentage of co-localised TERF1/PML foci are determined as percentages of telomeric foci that co-stained with PML over the total number of telomeric foci counted. **b**, **c**, **d**, **e**, **g** Data are presented as mean values ± SD. *indicates *p* < 0.05 and **indicates *p* < 0.005, Student’s *t*-test with two-tailed distribution. Source data are provided with this paper.

ALT is common in GBM in younger patient cohorts where it is highly correlated with *ATRX* mutations^6,7,9–11,24–26^. To identify additional factors which may promote ALT, we explored the cBioPortal database^27,28^ for mutations that overlapped with *ATRX*-mutant GBM in patients aged 30 years and younger^6,11^. We found that the majority of *ATRX*-mutated GBMs have additional mutations in the checkpoint regulator *TP53*, and either *H3F3A* (H3.3) or *IDH1* (Isocitrate Dehydrogenase 1) (Fig. 1a, Supplementary Table S1). The TP53 mutations were common across the *ATRX*-mutant GBMs, while H3.3 and IDH1 mutations were mutually exclusive. Three mutations were found in *H3F3A* (K27M, G34R and G34V), with G34R (*H3.3*^*G34R*^) being the most common^6^, while IDH1 mutations were almost exclusively IDH1^R132H^ (substitution of Arginine 132 with a Histidine). We, therefore, hypothesised that H3.3^G34R^ and IDH1^R132H^ are factors that collaborate with ATRX to activate ALT. To test this, we created mouse embryonic stem (ES) cell lines with homozygous knockouts of three genes known to be involved in ALT: *Atrx (-/Y)*, the checkpoint regulator *TP53 (−/−)*, and telomerase *Tert (−/−)* (APT-tKO), with and without a heterozygous H3.3^G34R^ substitution (H3.3^G34R^APT-tKO) (Supplementary Fig. 1). In the absence of telomerase activity, both the APT-tKO and H3.3^G34R^APT-tKO cell lines showed gradual erosion of telomeric DNA over time at a similar rate (Fig. 1b, c). Telomeres in APT-tKO cells were virtually undetectable by telomere DNA FISH analysis after 12 months in culture, and these cells reached a growth crisis after 16 months (Supplementary Fig. S2a, b). The H3.3^G34R^APT-tKO cells also reached a telomere crisis after 12 months in culture but recovered both cell growth and telomere length within 2 months (Fig. 1c, Supplementary Fig. S2a, b). As these cells are telomerase negative, the recovery of H3.3^G34R^APT-tKO cells was highly suggestive of ALT activation.

In support of ALT activation in our cell models, we detected ALT-associated hallmarks in H3.3^G34R^APT-tKO cells including telomeric C-circle DNA (Fig. 1d)^29^, sister chromatin exchange (Fig. 1e, Supplementary Fig. S2c; examples of TSCE are shown), and large ALT-associated promyelocytic leukaemia (PML) bodies (APBs) (Fig. 1f, g). The telomere recovery in H3.3^G34R^APT-tKO cells was also accompanied by the binding of TERF1, a Shelterin complex subunit (Fig. 1f, g). In contrast, TERF1 was undetectable in APT-tKO by 14 months, reflecting the loss of telomere repeats, and PML bodies remained as small, speckled and disorganised entities (Fig. 1f). These findings in H3.3^G34R^APT-tKO cells (H3.3^G34R^APT-tKO #1 line) were reproducible across an independent cell line (H3.3^G34R^APT-tKO #2; Supplementary Figs. S3 and S4). We note that all four mutations are required for activation of ALT, as single and double H3.3^G34R^ ^30^ and *Atrx*^*-/Y*^ ^31^ mutants, showed stable maintenance of telomere length over a period of 9 months although the H3.3^G34R^ *Atrx*^*-/Y*^ double mutant showed reduced telomere length (Supplementary Fig. S5). Together, these results suggest that H3.3^G34R^ is a cooperating mutation which acts in tandem with *ATRX*-KO to promote ALT.

### KDM4B is a key regulator of telomeric chromatin

**Fig. 2:**
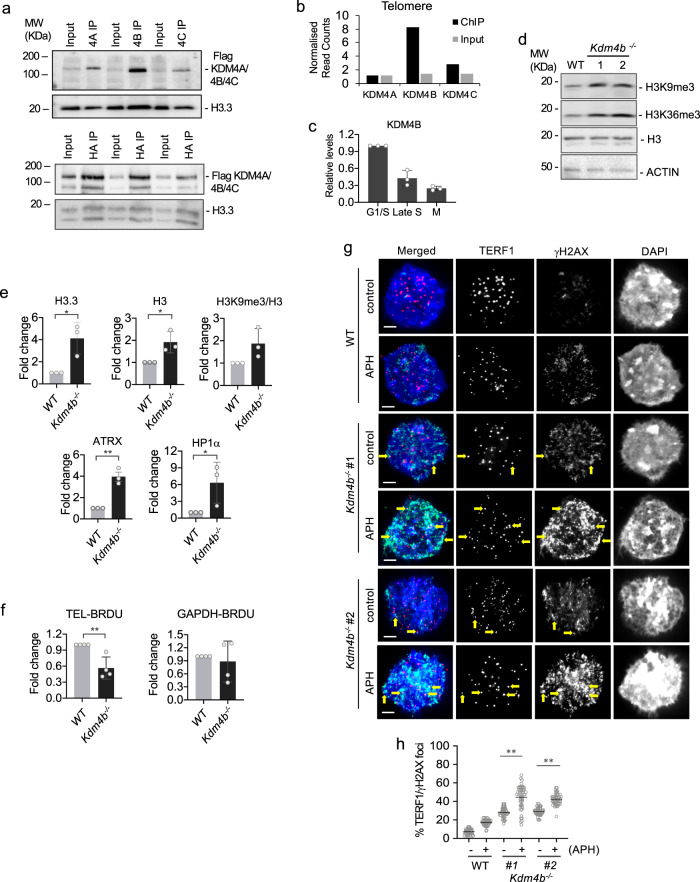
KDM4B^−/−^ mouse ES cells show replication stress. **a** Protein immunoprecipitation with an anti-Flag antibody in ES cells expressing HA-H3.3 and either Flag-tagged KDM4A, KDM4B or KDM4C, followed by western blot analysis with an anti-HA antibody. **b** ChIP-sequencing analysis of KDM4 -A^34^, -B^33^ and -C^33^ with input sequencing. Data shows reads which aligned to telomeres, normalised for total read counts showing KDM4B binding to telomeres. **c** ChIP-qPCR analysis of KDM4B at telomeres in cell cycle-synchronised cells (*n* = 3 independent experiments). **d** Western blot analyses of WT and KDM4B cells using antibodies against H3K9me3, H3K36me3, H3 and ACTIN. **e** ChIP-qPCR analyses of H3.3, total H3, H3K9me3, ATRX, HP1α. Level of H3K9me3 was normalised to total H3 levels (H3K9me3/H3) (*n* = 3 independent experiments). **f** ChIP-qPCR analyses of BrdU incorporation at telomeres and *Gapdh* promoter in WT and *Kdm4b*^*−/−*^ cells (*n* = 4 independent experiments). **g, h** Immunofluorescence analyses of TERF1 (red; diluted at 1/500) and γH2AX (green; diluted at 1/1500) in WT and *Kdm4b*^*−/−*^ cells with and without 1 mM APH treatment for 5 h. Arrows indicate presence of DNA damage (γH2AX) at telomeres (TERF1). Scale bars: 5 μm. Percentages of co-localised TERF1 and γH2AX foci in two independent *Kdm4b*^*−/−*^ cell lines (*Kdm4b*^*−/−*^ #1 and #2) are shown in **h**. For each cell line, >1500 telomeric or TERF1 foci were counted (*n* = 4 independent experiments). Percentage of co-localised TERF1/γH2AX foci are determined as percentages of telomeric foci that co-stained with γH2AX over the total number of telomeric foci counted. **c**, **e**, **f**, **h** Data are presented as mean values ± SD. *indicates *p* < 0.05 and **indicates *p* < 0.005, Student’s *t*-test with two-tailed distribution. **a**, **d**, **g** Representative images and blots from at least *n* = 3 independent experiments. Source data are provided with this paper.

We have previously shown that H3.3^G34R^ inhibits the KDM4 histone K9/K36 demethylases, leading to changes in H3K9me3 and H3K36me3 across the genome^30^. We, therefore, speculated that H3.3^G34R^ may facilitate ALT by inhibiting KDM4 function at telomeres. There are three major KDM4 enzymes in mammals; KDM4 -A, -B and -C, which all catalyse removal of H3K9 and H3K36 di- and trimethyl modifications^32^. Protein immunoprecipitation showed all three KDM4 proteins can interact with H3.3 (Fig. 2a) and chromatin immunoprecipitation-sequencing (ChIP-seq) data^33,34^ identified KDM4B as the most prominent demethylase at telomeres (Fig. 2b). KDM4B is also enriched at IAP-LTRs, relative to KDM4 A/C, while KDM4 A/C are preferentially enriched at GC-rich tandem repeats relative to KDM4B (Supplementary Fig. S6).

Previous studies have shown that KDM4A controls chromatin accessibility and alters replication timing of a late-replicating heterochromatic satellite region by demethylating H3K9me3 and antagonising the binding of heterochromatin protein 1 (HP1)^35,36^. Based on these findings, we hypothesised that KDM4B regulates accessibility of telomeric heterochromatin to facilitate DNA replication. Consistent with this, KDM4B is enriched at telomeres in G1/early S-phase (Fig. 2c). We created a *Kdm4b* homozygous knockout (*Kdm4b*^*−/−*^) mouse ES cell line (Supplementary Fig. S7a, b) and found increased total H3K9me3 and H3K36me3 in these cells (Fig. 2d). ChIP-qPCR of H3K9me3, ATRX, H3.3, total H3 and HP1α showed increased levels of all heterochromatin-associated proteins at telomeres in *Kdm4b*^*−/−*^ cells, suggestive of chromatin compaction and heterochromatinisation (Fig. 2e). This altered chromatin profile did not affect the maintenance of telomere length (Supplementary Fig. S7c) but led to increased replication stress at telomeres as detected by BrdU incorporation and a DNA damage marker (γH2AX) in two independent *Kdm4b*^*−/−*^ clones. *Kdm4b*^*−/−*^ cells showed decreased BrdU incorporation (Fig. 2f) as well as increased levels of γH2AX at the telomeres (Fig. 2g, h). Treatment with low concentrations of a DNA replication inhibitor, aphidicolin (APH), exacerbated this phenotype and led to substantially increased γH2AX at telomeres and across entire chromosomes (Fig. 2g, h, Supplementary Fig. S7d), while wild-type (WT) cells showed only modest differences. Of interest, *Kdm4b*^*−/−*^ cells also showed increased levels γH2AX at DAPI dense regions which are likely to be pericentric heterochromatin domains (Fig. 2g). Collectively, these results in our ES cell models show that KDM4B regulates chromatin accessibility, with its loss of function resulting in replication stress and damage at telomeres.

### Loss of ATRX and KDM4B function is required for ALT

ATRX has been proposed to resolve stalled replication forks through incorporation of H3.3, and much like the *Kdm4b*^*−/−*^ cells, loss of ATRX leads to increased DNA replication stress at telomeres^37^. It is possible that ATRX and KDM4B act in concert to maintain telomeres, such that ATRX can partially compensate for the loss of KDM4B, and vice versa. This would support the idea that H3.3^G34R^ promotes ALT through inhibition of KDM4B, as the H3.3^G34R^/*ATRX*-mutated GBMs would suffer a two-hit mutation in this pathway.

**Fig. 3:**
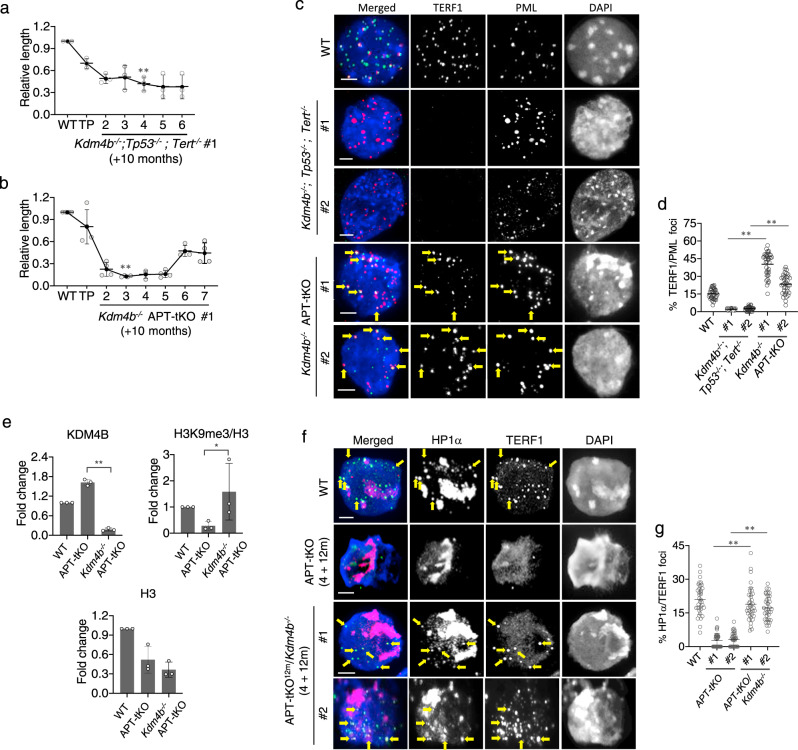
The inhibition of KDM4B by H3.3^G34R^ contributes to ALT activation. **a**, **b** Telomere length analyses of *Kdm4b*^*−/−*^*; Tp53*^*−/−*^*; Tert*^*−/−*^ and *Kdm4b*^*−/−*^APT-tKO cells over a 6-month period following knockout of *Kdm4b* in *Tp53*^*−/−*^*; Tert*^*−/−*^ (TP) and APT-tKO cells (*n* = 3 independent experiments). **c, d** Immunostaining of APBs in *Kdm4b*^*−/−*^*; Tp53*^*−/−*^*; Tert*^*−/−*^ and *Kdm4b*^*−/−*^APT-tKO cells, shown by co-staining of TERF1 (green) and PML (red). Arrows indicate co-staining of TERF1 and PML. Percentages of co-localised TERF1 and PML foci in *Kdm4b*^*−/−*^*; Tp53*^*−/−*^*; Tert*^*−/−*^ #1 and *Kdm4b*^*−/−*^APT-tKO #1 are shown in **d** (*n* = 5 independent experiments; >1200 foci counted for each line). **e** ChIP-PCR analyses of KDM4B, H3K9me3 and H3 in WT, APT-tKO and APT-tKO^12m^ *Kdm4b*^*−/−*^ cells. Level of H3K9me3 was normalised to total H3 levels (H3K9me3/H3) (*n* = 3 independent experiments). **f, g** Immunostaining of TERF1 (green; diluted at 1/500) and HP1α (red; diluted at 1/500) in WT, APT-tKO^12m^ and APT-tKO^12m^ *Kdm4b*^*−/−*^ cells, 4 months in culture following *Kdm4b* knockout. Arrows indicate co-staining of TERF1 and HP1α. Percentages of co-localised TERF1 and HP1α foci in APT-tKO^12m^, APT-tKO^12m^ *Kdm4b*^*−/−*^#1 and #2 are shown in **g**. For each cell line, >1500 telomeric or TERF1 foci were counted (*n* = 3 independent experiments). Percentage of co-localised TERF1/HP1α foci are determined as percentages of telomeric foci that co-stained with HP1α over the total number of telomeric foci counted. **a**, **b**, **d**, **e**, **g** Data are presented as mean values ± SD. *indicates *p* < 0.05 and ** indicates *p* < 0.005, Student’s *t*-test with two-tailed distribution. **c**, **f** Scale bars: 5 μm; representative images from at least *n* = 3 independent experiments. Source data are provided with this paper.

To test if H3.3^G34R^ is activating ALT through inhibition of KDM4B, we knocked out KDM4B directly in APT-tKO (*Kdm4b*^*−/−*^APT-tKO) cell lines that had been cultured for 10 months (Supplementary Fig. S8). In order to determine if KDM4B and ATRX acted in concert, we also knocked out *Kdm4b* in *Tp53*^*−/−*^*; Tert*^*−/−*^ cells with WT ATRX (*Kdm4b*^*−/−*^*; Tp53*^*−/−*^*; Tert*^*−/−*^) (Supplementary Fig. S8). Both lines showed a gradual loss of telomeric DNA over time, with a more pronounced loss in the *Kdm4b*^*−/−*^APT-tKO cells (Fig. 3a, b, Supplementary Fig. S9). Cells with intact ATRX (*Kdm4b*^*−/−*^*; Tp53*^*−/−*^*; Tert*^*−/−*^) reached a growth crisis after a further 6 months in culture, and telomeres were undetectable by either FISH or TERF1 immunostaining (Fig. 3c, Supplementary Fig. S9a, b). In contrast, telomeres in *Kdm4b*^*−/−*^APT-tKO cells shortened rapidly over a 4-month period, followed by recovery of telomere length and cell growth by 6 months (Fig. 3b, Supplementary Fig. S9a, b). Much like the H3.3^G34R^APT-tKO cells, the *Kdm4b*^*−/−*^APT-tKO cells showed all the hallmarks of ALT, namely recovery of telomere length, restored TERF1 binding and telomeres encapsulated by APBs (Fig. 3c, d). These findings in *Kdm4b*^*−/−*^APT-tKO cells (*Kdm4b*^*−/−*^APT-tKO #1) were reproducible in an independent cell line (*Kdm4b*^*−/−*^APT-tKO #2; Supplementary Fig. S10). Taken together, these results demonstrate that a combinatorial loss of KDM4B and ATRX is required to activate ALT, and support the idea that H3.3^G34R^ acts through inhibition of KDM4B.

Unlike the H3.3^G34R^APT-tKO and *Kdm4b*^*−/−*^APT-tKO, the APT-tKO cells with functional KDM4B were unable to activate ALT (Fig. 1b) and failed to form the large APBs at telomeres which are required for ALT (Fig. 1f, g). Despite the presence of PML bodies, APT-tKO cells failed to form large APBs with telomeric co-localisation and the telomere signals were below the level of detection, whereas H3.3^G34R^APT-tKO and *Kdm4b*^*−/−*^APT-tKO (Fig. 1f, g, 3c, d, Supplementary Fig. S4a) form APBs following ALT activation. PML bodies are phase-separated liquid compartments stabilised by multivalent interactions between proteins and nucleic acids^38^ and function in heterochromatin remodelling^39–41^. The heterochromatin protein, HP1α, is known to drive phase separation^42,43^ and HP1α binds the H3K9me3 residue^44^ which is demethylated by KDM4B. It is therefore possible that the continued activity of KDM4B at APT-tKO telomeres prevents the formation of phase-separated APBs by reducing HP1α recruitment through the removal of H3K9me3. Consistent with this, we found that APT-tKO cells had elevated KDM4B binding at telomeres and reduced H3K9me3 (Fig. 3e). To test if removal of KDM4B was sufficient to nucleate the formation of APBs, we knocked out *Kdm4b* in late-passage (12 months continuous culture) APT-tKO cells (APT-tKO^12m^) with telomeres in prolonged crisis (Supplementary Fig. S11a, b). The acute removal of KDM4B in APT-tKO^12m^ (APT-tKO^12m^/*Kdm4b*^*−/−*^) led to increased H3K9me3 and HP1α at telomeres (Fig. 3e, f, g), accompanied by formation of APBs and restoration of TERF1 binding (Supplementary Fig. S11c, d). It is clear that the loss of ATRX is an absolute requirement for ALT, yet, paradoxically, loss of ATRX alone (APT-tKO) creates a chromatin environment that precludes the formation of APBs at telomeres. ALT telomere maintenance requires a threshold level of heterochromatin^45–47^ which in this case was achieved through inactivation of KDM4B. Our results suggest that in *ATRX*-mutated ALT cancers, loss of KDM4B results in an increase of H3K9me3- and HP1α-containing heterochromatin to a level that can sustain and stabilise the formation of APBs.

### KDM4B activity inhibits APB formation and the ALT pathway

**Fig. 4:**
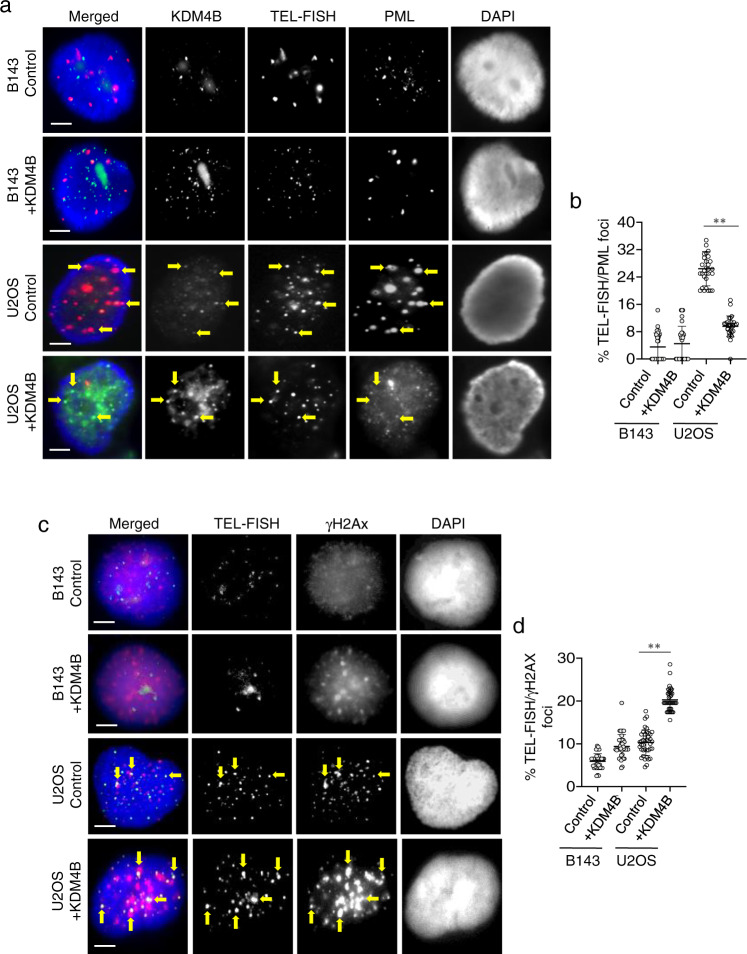
KDM4B activity inhibits the ALT pathway. Immunostaining of KDM4B (green), PML (red) and TEL-FISH (cyan) in control or KDM4B transfected B143 and U2OS cells. Arrows show KDM4B at telomeres (**a**). Percentages of co-localised TEL-FISH and PML foci in H3.3^G34R^APT-tKO#1 cells are shown in **b** (*n* = 3 independent experiments; >500 foci counted). Immunostaining of γH2AX (red; diluted at 1/500) and TEL-FISH (green) analyses in B143 and U2OS cells with and without overexpression of KDM4B (+KDM4B). Arrows show γ-H2AX at telomeres (**c**). Percentages of co-localised TEL-FISH and γH2AX foci are shown in **d**. For each cell line, >1500 telomeric or TEL-FISH telomeric foci were counted (*n* = 4 independent experiments). Percentage of co-localised TEL-FISH/γH2AX foci are determined as percentages of telomeric foci that co-stained with γH2AX over the total number of telomeric foci counted. **b**, **d** Data are presented as mean values ± SD. **indicates *p* < 0.005, Student’s *t*-test with two-tailed distribution. **a**, **c** Scale bars: 5 μm; representative images from at least *n* = 3 independent experiments. Source data are provided with this paper.

If loss of KDM4B activity is critical for initiating ALT, then expression of KDM4B would likely be detrimental to established ALT cell lines. We tested this by overexpression of KDM4B in two human ALT cell lines (U2OS and GM847), alongside two non-ALT counterparts (B143, HEK293). In ALT-positive cells, U2OS (Fig. 4a–d) and GM847 (Supplementary Fig. S12a–d), ectopic expression of KDM4B led to prominent binding of KDM4B at telomeres (Fig. 4a, b; Supplementary Fig. S12a, b) accompanied by loss of APBs and high levels of DNA damage (γH2AX) (Fig. 4c, d; Supplementary Fig. S12c, d). In contrast, ectopic expression of KDM4B in non-ALT counterparts, B143 (Fig. 4a–d) and HEK293 (Supplementary Fig. 12a–d) showed only low levels of KDM4B binding (Fig. 4a, b; Supplementary Fig. 12a, b) with no significant increase of γH2AX (Fig. 4c, d; Supplementary Fig. S12c, d) at the telomeres. These results demonstrate that expression of KDM4B is sufficient to disrupt APBs and induce telomeric damage in an established ALT cell model.

**Fig. 5:**
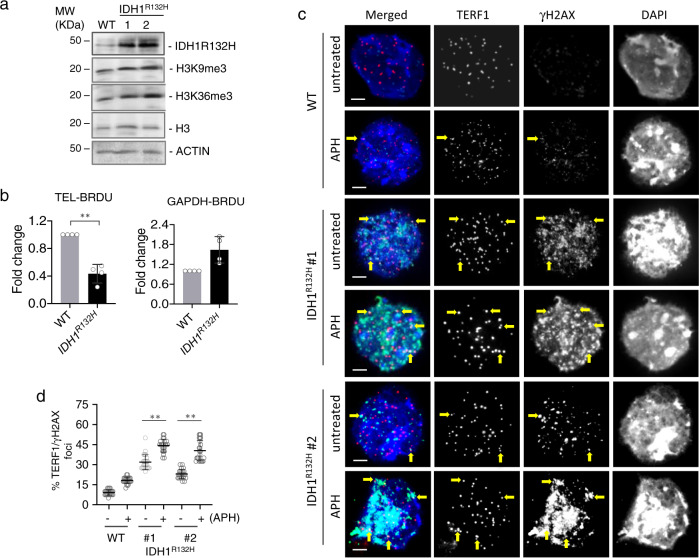
IDH1^R132H^ mouse ES cells show replication stress. **a** Western blot analyses of WT and IDH1^R132H^ ES cells using antibodies against IDH1R132H, H3K9me3, H3, H3K36me3 and ACTIN. **b** IP-qPCR analyses of BrdU incorporation at telomeres and *Gapdh* promoter in WT and IDH1^R132H^ ES cells (*n* = 3 independent experiments). Immunofluorescence analyses of TERF1 (red; diluted at 1/500) and γH2AX (green; diluted at 1/1500) in WT and IDH1^R132H^ ES cells with and without 1 mM APH treatment for 5 h. Arrows indicate co-staining of γH2AX and TERF1 (**c**). Percentages of co-localised TERF1 and γH2AX foci are shown in **d**. For each cell line, >1500 telomeric or TERF1 foci were counted (*n* = 4 independent experiments). Scale bars: 5 μm. Percentage of co-localised TERF1/γH2AX foci are determined as percentages of telomeric foci that co-stained with γH2AX over the total number of telomeric foci counted. **b**, **d** Data are presented as mean values ± SD. **p < 0.005, Student’s *t*-test with two-tailed distribution. **a**, **c** Representative images and blots from at least *n* = 3 independent experiments. Source data are provided with this paper.

In addition to H3.3^G34R^, ATRX-mutated ALT-positive GBMs are also frequently mutated in IDH1 (IDH1^R132H^), and these two mutations are mutually exclusive (Fig. 1a). The IDH1^R132H^ mutation produces a 2-hydroxyglutarate oncogenic metabolite which inhibits 2-oxo-glutarate dependent di-oxygenases, including the KDM4 family of enzymes^48^. It is therefore possible that, like the H3.3^G34R^ mutations, the IDH1^R132H^ mutation also promotes ALT through inhibition of KDM4B. We created mouse ES cells with an IDH1^R132H^ mutation (Supplementary Fig. S13a) and observed many similarities with *Kdm4b*^*−/−*^ cells, supporting the idea that IDH1^R132H^ inhibits KDM4B. The IDH1^R132H^ mutants (clones #1 and #2) showed increased levels of total H3K9me3 and H3K36me3 (Fig. 5a) with no significant change in telomere length (Supplementary Fig. S13b, c). The IDH1^R132H^ mutants also showed a telomere stress phenotype very similar to *Kdm4b*^*−/−*^ cells including decreased BrdU incorporation (Fig. 5b), increased γH2AX at telomeres, and increased sensitivity to APH treatment (Fig. 5c, d Supplementary Fig. S13d). In addition, much like the *Kdm4b*^*−/−*^ mutant, the IDH1^R132H^ mutants also showed increased staining of γH2AX at DAPI dense regions which are likely the pericentric heterochromatin. These results show that IDH1^R132H^ induces a telomere replication stress phenotype similar to that seen in *Kdm4b*^*−/−*^ cells. This strongly suggests that both H3.3^G34R^ and IDH1^R132H^ can inhibit KDM4B activity, and that inhibition of KDM4B is a common component in promoting ALT in *ATRX*-mutated cancers.

## Discussion

ALT tumours are frequently inactivated for ATRX, yet inherited mutations in *ATRX* (underlying the ATR-X syndrome) do not have appreciable effects on cancer predisposition. The loss of ATRX is therefore necessary but not sufficient for ALT, and additional events are required for ALT activation. By searching public cancer genome databases, we identified H3.3^G34R^ and IDH1^R132H^ as potential mutations which may collaborate with ATRX to induce ALT in glioblastomas affecting younger age groups. By creating primary cell models of ALT using mouse ES cells, we discovered that combined mutations of H3.3^G34R^ and ATRX are required to activate ALT, and H3.3^G34R^ promotes ALT by inhibiting the KDM4B histone demethylase at telomeres. We demonstrate in mouse ES cells that H3.3^G34R^ and *Kdm4b*^*−/−*^ are functionally equivalent in promoting ALT, and it is highly likely that IDH1^R132H^ acts in the same pathway. Furthermore, ectopic expression of KDM4B induces telomere damage in ALT-positive cells. Our data suggest that loss of KDM4B is required for ALT, and provides the first example of ALT activation in a primary cell model by manipulating four defined factors: inactivation of telomerase, TP53 checkpoint, ATRX, and KDM4B (either H3.3^G34R^ or *Kdm4b*^*−/−*^).

The maintenance of telomeres in ALT requires many DNA repair proteins^12,13^ and this complex process is dependent on the formation of phase-separated APBs at ALT telomeres^5,14,15^. APBs are one of the defining hallmarks of ALT and removal of APBs in ALT cancer lines results in telomere shortening and damage^49,50^. In addition, the coalescence of APB condensates may promote telomere clustering/recombination which is required for telomere DNA synthesis in ALT^14,51,52^. As telomeres are heterochromatic, formation of APBs is likely dependent on HP1α which is known to be a strong driver of phase separation^42,43^. The binding of HP1α is dependent on H3K9me3^44^, the modification demethylated by KDM4B. In normal cells, KDM4B most likely plays a role in controlling chromatin accessibility by antagonising H3K9me3/HP1α at telomeres^36^. This appears to be particularly important for facilitating DNA replication, as knockout (*Kdm4b*^*−/−*^) or inhibition (IDH1^R132H^) of KDM4B leads to increased replication stress and DNA damage at telomeres. However, in ALT cells the continued demethylase activity of KDM4B at telomeres could become a liability in the absence of ATRX-mediated heterochromatin assembly, as further losses of H3K9me3/HP1α could create a chromatin environment incompatible with formation of APBs. Supporting this, ectopic expression of KDM4B in established ALT cancer lines leads to dissociation of APBs and increased DNA damage at telomeres. Conversely, inhibition (H3.3^G34R^, IDH1^R132H^) or knockout (*Kdm4b*^*−/−*^) of KDM4B leads to increased H3K9me3 and HP1α at telomeres and facilitates APB formation in our mutant cell lines.

However, we note a quiescent period occurring in between the telomere crisis, and the nucleation of APBs and telomere lengthening in our cell models. This period likely represents the initiation phase of ALT which may include events such as the recruitment of factors, and execution of processes that stabilise and prepare telomeres for lengthening. To date, most investigations have been conducted on ALT cell lines derived from advanced cancers when the process of ALT is well underway. As a result, very little is known about the initiation and early phases of ALT. As we have now established a cell model which reliably undergoes ALT, it should now be possible to investigate these early stages in more detail. In addition, we cannot exclude the possibility that additional factors may be involved in activating ALT during telomere crisis. For example, loss of telomerase may be directly required for ALT activation. While previous studies in human cancer cell lines have shown that expression of telomerase (*TERT*) does not abolish ALT activity^53^, inhibition of telomerase in a mouse cancer model can induce ALT^54^. Furthermore, a number of studies have reported the non-canonical role of telomerase in telomeric chromatin maintenance and resolution of DNA damage, in addition to its function in telomere DNA elongation^55,56^. It remains to be clarified how telomerase loss may contribute to ALT, and if it suppresses ALT by removing telomeric intermediates that arise during telomeric replication stress or crisis.

We have identified KDM4B as a novel factor involved in ALT in *ATRX*-mutated glioblastomas, and KDM4B can be inactivated either through H3.3^G34R^ or IDH1^R132H^. Intriguingly, *ATRX* mutations and ALT are also common in paragangliomas and pheochromocytomas^57,58^, and these tumours have frequent mutations in Krebs cycle genes, such as fumarate hydratase (*FH*) and succinate dehydrogenase (*SDHx*), leading to an accumulation of fumarate and succinate^32,58,59^. Much like the IDH1/2 mutations, fumarate and succinate are competitive inhibitors of α-KG-dependent dioxygenases, including KDM4B^32,59^. It will be interesting to investigate if *FH* and *SDHx* mutations also inhibit KDM4B and act as cooperating factors to promote ALT in *ATRX*-mutated cancers. In conclusion, we provide evidence that inactivation of KDM4B is a critical factor in activating the ALT pathway in ATRX-mutated glioblastomas. The clinical significance of KDM4B inactivation in other ATRX-mutated ALT cancers requires further studies.

## Methods

### Gene mutation analysis

Information regarding *ATRX*, *TP53*, *H3F3A* and *IDH1/2* alterations in human GBM tumours (Supplementary Table S1) was obtained from The Cancer Genome Atlas (TCGA) database, an open-access database publicly available at http://www.cbioportal.org^6,11,26–28,60,61^.

### Cell culture

Mouse ES cells and derived cell lines were cultured in Dulbecco’s modified Eagle’s medium supplemented with 15% heat-inactivated foetal calf serum, 10^3^ units/ml leukaemia inhibitory factor (Merck), 0.1 mM β-mercaptoethanol, non-essential amino acids, L-glutamine and penicillin/streptomycin. Cells were maintained in 37 °C incubator under 5% CO_2_. Human B143, HEK293, GM847 and U2OS cells were cultured in Dulbecco’s modified Eagle’s medium supplemented with 10% heat-inactivated foetal calf serum and penicillin/streptomycin.

### Generation of knockout and mutant ES cell lines

The H3.3^G34R^ and *Atrx*^*-/Y*^ ES cell lines were generated in our previous studies^30,31^. CRISPR-Cas9 mediated *Atrx, Tp53, Tert and Kdm4b* knockouts were carried out using specific guide RNA (gRNA) (Supplementary Table S2). All positive targeted ES cell lines were identified by PCR analyses using specific primer sets (Supplementary Table 2), followed by confirmation either by reverse transcription-qPCR or Western blot analysis.

Targeted deletion of *Atrx* exon 17 using *Atrx* RNA guide #1 and #2 was screened by identification of a truncation of a 1850 bp PCR product amplified with Atrx ex17 For and Rev primers.

Targeted deletion of *Tp53* exon 4 using *Tp53* RNA guide #1 and #2 was identified by a reduction of a 340 bp PCR product amplified with Tp53 ex4 For and Rev primers.

Targeted deletion of *Tert* exon 2 using *Tert* RNA guide #1 and #2 was identified by a truncation of a 1048 bp PCR product amplified with Tert ex2 g2F and g1R primers.

Targeted deletion of *Tert* exon 2 using *Tert* RNA guide #4 and #5 was identified by a reduction of a 1252 bp PCR product amplified with Tert ex2 g4F and g5R primers.

Targeted deletion of *Kdm4b* exon 3 and 5 using *Kdm4b* RNA guides #1 and #2 was identified by a loss of a 503 bp PCR product amplified using Kdm4b ex 3 F and 3 R primers.

IDH1^R132H^ mutation was introduced by transfecting *Idh1* ex3 guide 1 (targeting exon 3 of *Idh1*) together with a repair DNA template carrying a R132H substitution (Supplementary Table S2). Positive mutant lines were screened by PCR amplification of a 604 bp PCR product with Idh1 ex3 For and Rev primers, followed by restriction digest with *EcoRI* to generate 423 and 181 bp fragments.

### Doubling time calculation

In brief, 10^5^ cells were plated on a 60 mm dish; after 28 hours, the cells were counted. The population doubling time or the time required for a culture to double in number is calculated with the following formula:

DT = T ln2/ln(Xe/Xb)

*T is the incubation time in any units*.

*Xb is the cell number at the beginning of the incubation time*.

*Xe is the cell number at the end of the incubation time*.

### Antibodies

Antibodies used were directed against anti-H3 (Abcam, #ab1791), anti-H3.3 (Abcam, #ab176840), anti-H3K9me3 (Abcam, ab8898), anti-H3K36me3 (Abcam, #ab9050), anti-γH2A.X/phospho-histone H2A.X (Ser139) clone JBW301 (Merck Millipore, #05-636), anti-ATRX (Santa Cruz Biotechnologies, #sc15408), anti- KDM4B (Abcam, #ab191434) anti-IDH1 (Sigma Aldrich, SAB4100064), anti-IDH1^R132H^ (Sigma Aldrich, SAB4200548), anti-HP1α (Merck Millipore, #MAB3584), anti-PML (Merck Millipore, #MAB3738), anti-TP53 (Cell Signaling Technologies, #cst-2524), anti-Flag (Sigma, #F1804), anti-TERF1 (Alpha Diagnostics, #TRF12-S) and anti-BrdU (Abcam, #ab6326), Anti-β Actin (AC-15) (Santa Cruz Biotechnology, #sc69879), Goat anti Rabbit IgG, HRP conjugate (Merck Millipore, #AP187P), Donkey anti-Mouse IgG HRP conjugate (Merck Millipore, #AP192P), Donkey anti-Mouse IgG (H + L) Alexa Fluor 594 (Invitrogen, #A-21203), Donkey anti-Rabbit IgG (H + L) Alexa Fluor 488 (Invitrogen, #A-21206).

### Immunofluorescence

Cells were treated with KaryoMAX Colcemid at a final concentration of 10 μg/ml for 1 h prior to harvest. Cells were subjected to hypotonic treatment in 0.075 M KCl (room temperature for 5 min), cytospun onto slides, and incubated in ice-cold KCM buffer (120 mM KCl, 10 mM Tris-HCl pH 7.5, 20 mM NaCl, 0.5 mM EDTA, 0.1% (v/v) Triton X-100 and protease inhibitor) for 5 min. Slides were incubated in ice-cold KCM extraction buffer (KCM and 0.4% Triton X-100) for 5 min, followed by an incubation in ice-cold KCM blocking buffer (KCM, 2% BSA, protease inhibitor and AEBSF) for another 5 min. Slides were incubated in the relevant primary (diluted in 1/250 to 500) and secondary antibodies (diluted in 1/1200) for 1 h at 37 °C in KCM block buffer. After each antibody incubation, slides were washed three times with ice-cold KCM. After washing, they were fixed in 4% (v/v) formaldehyde (in KCM) and mounted with DAPI in Vectashield media. Images were collected using a Zeiss imager M2 fluorescence microscope with 100X objective linked to an AxioCam MRm CCD camera system. Images were collected using Zeiss Zen Imaging Software. To determine the percentages of co-localised TERF1/PML, TERF1/HP1α or TERF1/γH2AX foci, at least 1200–1500 telomeric foci were counted over three independent experiments for each cell line. Percentages of co-localised TERF1/PML, TERF1/HP1α or TERF1/γH2AX foci represent percentages of telomeric foci that show co-staining of PML, HP1α or γH2AX over the total number of telomeric foci counted in respective experiments.

### Fluorescence in-situ hybridisation (FISH)

Cells were treated with Colcemid (KaryoMAX) at a final concentration of 10 μg/ml for 1 h at 37 °C prior to harvesting. Cells were subjected to hypotonic treatment in 0.075 M KCl, fixed in methanol: acetic acid (3:1 ratio), and pelleted for 5 min at 900 *g*. The cells were washed with fixative two more times, followed by centrifugation prior to each wash. The fixed cells were dropped onto slides, then dehydrated sequentially in a 75%, 85%, and 100% ethanol series at room temperature for 5 min each. Slides were rehydrated in 1× phosphate buffer saline (PBS) (137 mM NaCl, 5.4 mM KCl, 10 mM Na_2_HPO_4_, and 5 mM NaH_2_PO_4_H_2_O) at room temperature for 5 min and incubated with 0.2 μg/ml RNase A in 1 × PBS for 30 min at 37 °C. Slides were washed once in 1× PBS, fixed in 4% PFA (in PBS), washed again in 1 × PBS, and lastly ethanol dehydrated for 5 min each. FISH was performed by hybridisation with telomeric and centromeric DNA probes (PNA bio) in hybridisation buffer (20 mM Tris-HCl pH 7.5, 10 mM NaHPO_4_ pH 7.4, 10 mM NaCl, and 50% formamide) and 30 μg salmon sperm DNA (Invitrogen) for 3 min at 80 °C and left to incubate overnight at 37 °C. Slides were washed in 2× SSC at room temperature followed by three rounds of washes at 50 °C in 0.5× SSC. Slides were ethanol-dehydrated at room temperature for 5 min, left to air dry, and mounted with DAPI in Vectashield mounting media. Images were collected as described above.

### CO-FISH

Cells were incubated for 12–14 h in fresh media (DMEM) containing BrdU (10 μg/ml) and BrdC (3.33 μg/ml). An hour before harvesting cells were treated with KaryoMAX Colcemid at a final concentration of 10 μg/ml. Cells were subjected to hypotonic treatment in 0.075 M KCl, fixed in methanol: acetic acid (3:1 ratio), and pelleted at 900 g for 5 min. The cells were washed with fixative two more times, with centrifugation between washes. The fixed cells were dropped onto slides and dehydrated in a 75%, 85%, and 100% ethanol series at room temperature for 5 min each and left to air dry. Slides were rehydrated in 1 × PBS at room temperature for 5 min, incubated with 0.2 μg/ml RNase A (in PBS) at 37 °C for 30 min, and stained with 0.5 μg/ml Hoechst 33258 dye (in 2× saline sodium citrate (SSC: 150 mM NaCl, 15 mM sodium citrate, pH 7) at room temperature for 15 min (in the dark). Slides were subsequently placed into a shallow tray, covered with 2× SSC, and exposed to 365 nm ultraviolet light for 45 min. The BrdU/C incorporated strands were digested with at least 10 U/μl Exonuclease III at room temperature for 1 h. Slides were washed in 1× PBS, dehydrated in an ethanol series, and left to air dry. FISH was performed by hybridisation with a TAACCC and TTAGGG telomere peptide nucleic acid probe in hybridisation buffer (20 mM Tris-HCl pH 7.5, 10 mM NaHPO4, pH 7.4, 10 mM NaCl and 50% formamide) and 30 μg salmon sperm DNA (Invitrogen) overnight at 37 °C (non-denaturing). Slides were washed in 2× SSC at room temperature followed by three rounds of washes at 50 °C in 0.5× SSC. Slides were ethanol-dehydrated at room temperature for 5 min, left to air dry, and mounted with DAPI in Vectashield mounting media. Images were collected as described above.

### Real time-qPCR analysis of gene expression

RNA was prepared using High Pure RNA Isolation kit according to the manufacturer’s protocol (Roche). cDNA was synthesised using the cDNA Reverse Transcriptase kit (Life Technologies). The expression levels of target genes were quantitated by qPCR with FastStart Essential DNA Green Master (Roche) using the LightCycler and analysed with LightCycler^®^96 Software (Roche Life Science). As an internal control, primers specific for *Gapdh* were used in real-time PCR analysis. The comparative cycle threshold method was used for data analyses and relative fold difference was expressed as 2−ΔΔCT.

### Chromatin immunoprecipitation (ChIP)

Cells were harvested and crosslinked with 1% paraformaldehyde for 10 min at room temperature. For ATRX and KDM4B ChIP, cells were cross-linked first with 2 mM EGS (Pierce 26103) for 45 min then with 1% paraformaldehyde for 20 min. Excess formaldehyde was quenched with glycine at a final concentration of 0.25 M. Cell were washed with PBS, pelleted and lysed in cold cell lysis buffer (10 mM Tris pH 8, 10 mM NaCl, 0.2% NP40 and protease inhibitors). Nuclei were centrifuged and resuspended in 50 mM Tris pH 8, 10 mM EDTA and 1% sodium dodecyl sulphate (SDS), and sonicated with a Bioruptor (Diagenode) to obtain chromatin fragments of 500 bp or less. Resulted chromatin was diluted in IP dilution buffer (20 mM Tris pH 8, 2 mM EDTA, 150 mM NaCl, 1% Triton X-100 and 0.01% SDS and protease inhibitors) and pre-cleared with Protein A Agarose beads at 4 °C. Pre-cleared chromatin was immunoprecipitated with antibody-bound beads at 4 °C overnight. For each ChIP reaction, 2–5 μg of antibody and 30 μl of Protein A Agarose beads (50% slurry) were used. The immunoprecipitated material was washed and eluted in 100 mM NaHCO_3_ and 1% SDS. The eluted material was treated with RNaseA, Proteinase K and NaCl, and reverse-crosslinked at 65 °C overnight. 10 μg tRNA and equal volume Tris-EDTA buffer were added and DNA was phenol/chloroform extracted, followed by precipitation using tRNA and glycogen as carriers (5 μg). Purified ChIP DNA was used as template for qPCR using the primers corresponding to telomeric repeats (Telomere Forward and Reverse) and *Gapdh* promoter (Gapdh Pro For and Rev).

### ChIP-seq data

Fastq files of KDM4A^34^, KDM4B^33^ and KDM4C^33^ ChIP-seq and matched input samples were aligned to a repeat database with Repeat Enrichment Estimator v1.0^62^. In brief, a repeat assembly file was generated using the Repbase database and reads were aligned to this library and counted. Further details are available in reference^62^. Reads which aligned to telomeres (annotated as (CCCTTA)n in the mouse repeats database) are shown after normalising for total read counts.

### BrdU ChIP

Cells were treated with 10 µg/mL BrdU (Sigma) overnight before harvesting. Genomic DNA was extracted by Qiagen DNeasy kit. gDNA was then sheared into 100–300 bp fragments using a probe sonicator. 10 μg of sheared gDNA was denatured for 10 min at 95 °C and cooled on ice. Denatured gDNA was incubated with 2 μg anti-IgG (Sigma) or anti-BrdU (Abcam, ab6326) antibody diluted in immunoprecipitation buffer (0.0625% (v/v) Triton X-100 in PBS), followed by constant rotation overnight at 4 °C. The next day, samples were incubated with 30 μl Protein G magnetic beads (Pierce) for 2 h rotating at 4 °C. Beads were subsequently washed three times with immunoprecipitation buffer and once with TE buffer. Beads were then incubated twice in elution buffer (1% (w/v) SDS in 1 × TE buffer) for 15 min at 65 °C. DNA was precipitated from pooled eluates, along with 10% inputs, with 300 mM Sodium Acetate (pH 5.2) and Ethanol. Immunoprecipitated DNA was quantified by qPCR using Telomere primers. A standard curve was plotted by serial dilution of Input DNA.

### qPCR analysis of telomere Length

Genomic DNA was prepared from mouse ES cell lines for real-time quantitative PCR analysis of telomere length^63,64^ using the LightCycler and analysed with LightCycler^®^ 480 Software. The average telomere length was measured by quantifying telomeric DNA relative to a single-copy gene. 36B4 was used as the single-copy reference gene. 2 ng of genomic DNA, 300 nM primers, and DNA SYBR Green PCR Master mix (Roche) were used for the qPCR reactions. For telomere DNA amplification, the PCR cycling parameters used were 95 °C for 10 min, 30 cycles of 95 °C for 15 sec and a 56 °C anneal-extension step for 1 min. For 36B4 DNA amplification, the PCR cycling parameters used were 95 °C for 10 min, 35 cycles of 95 °C for 15 s, 52 °C annealing for 20 s and 72 °C extension for 30 s. A standard curve was set up for each primer set using wildtype genomic DNA template over a range of 0.8 ng to 100 ng. Relative telomere length was represented by the value of telomere divided by the value of *36B4* gene.

### Telomeric C-circle/qPCR assay

Telomeric C-circle was detected using an assay previously described by Lau et al. 2013^65^. Rolling circle amplification of C-circle was performed with 40 ng of genomic DNA, 0.2 µg/µL BSA, 0.1% Tween, 4 mM DTT, 1.2 mM each of dATP, dCTP, dGTP, dTTP and φ29 DNA polymerase (φ29; 3.75 U/16 ng DNA) (NEB) in 1× φ29 buffer). The reaction was incubated at 30 °C for 8 h then at 70 °C for 20 min. For each sample, the assay was performed with and without φ29. The assay products were diluted for qPCR analyses using telomeric DNA and single-copy gene primer (*36B4*) to determine the level by measuring the increase in total telomeric DNA. C-circle level was reported as the relative telomeric content of the C-circle assay products relative to a single-copy gene (*36B4*).

### Reporting summary

Further information on research design is available in the Nature Research Reporting Summary linked to this article.

## Supplementary information

Supplementary Information

Reporting Summary

## Data Availability

The data that support this study are available from the corresponding author upon reasonable request. Source data are provided with this paper.

## Source data

Source Data
