# Supplementary material for: Mutations inhibiting KDM4B drive ALT activation in ATRX-mutated glioblastomas
Source: Nat Commun. 2021 May 10;12:2584. doi: 10.1038/s41467-021-22543-z (PMC8110556; doi:10.1038/s41467-021-22543-z)
Supplement: Supplementary file 1 — Supplementary Information [file 41467_2021_22543_MOESM1_ESM.pdf]

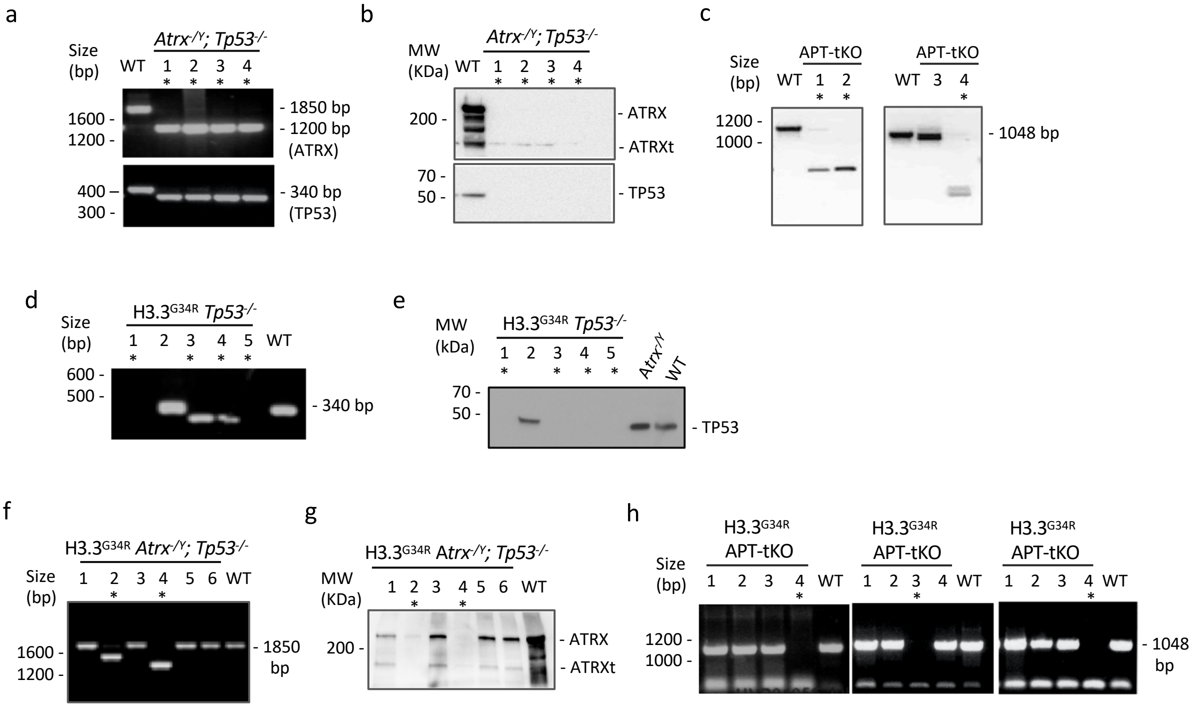

Supplementary Fig 1. Generation of *Atrx*$^{-/Y}$;*Tp53*$^{-/-}$, *Tert*$^{-/-}$ and H3.3^{G34R} APT-tKO mouse ES cells.

(**a, b)** Generation of *Atrx*$^{-/Y}$; *Tp53*$^{-/-}$ cells. **(a)** CRISPR-Cas9–mediated DNA editing system was used to knock out the *Atrx* and *Tp53* genes in wild-type (WT) mouse ES cells. Deletion of *Atrx* exon 17 using *Atrx* RNA guide #1 and #2 led to a truncation of a 1850 bp PCR product to 1200 bp amplified with Atrx ex17 For and Rev primers (top panel). Deletion of *Tp53* exon 4 using *Tp53* RNA guide #1 and #2 resulted in a reduction of a 340 bp PCR product amplified with Tp53 ex4 For and Rev primers (bottom panel). **(b)** Western blot analysis showing losses of ATRX (275 KDa) and TP53 (53 kDa) proteins in *Atrx*$^{-/Y}$; *Tp53*$^{-/-}$ cells. The faint truncated ATRX isoform (190 kDa) is also shown. * indicates positive *Atrx*$^{-/Y}$; *Tp53*$^{-/-}$ clones. **(c)** Generation of APT-tKO (*Atrx*$^{-/Y}$; *Tp53*$^{-/-}$; *Tert*$^{-/-}$) cells. Targeted deletion of *Tert* exon 2 using *Tert* RNA guide #1 and #2 in *Atrx*$^{-/Y}$; *Tp53*$^{-/-}$ cells led to a reduction of a 1048 bp PCR product amplified with Tert ex2 g2F and g1R primers. * indicates positive APT-tKO clones. **(d, e)** Generation of H3.3^{G34R} *Tp53*$^{-/-}$ cells. **(d)** CRISPR/Cas9–mediated deletion of *Tp53* exon 4 using *Tp53* RNA guide #1 and #2 in H3.3^{G34R} cells resulted in a reduction of a 340 bp PCR product amplified with Tp53 ex4 For and Rev primers. **(e)** Western blot analysis showing a loss of TP53 protein (53 KDa). * indicates positive H3.3^{G34R} *Tp53*$^{-/-}$ clones. (**f, g)** Generation of H3.3^{G34R} *Atrx*$^{-/Y}$; *Tp53*$^{-/-}$ cells. **(f)** Deletion of *Atrx* exon 17 using *Atrx* RNA guide #1 and #2 in H3.3^{G34R} *Tp53*$^{-/-}$ cells resulted in a truncation of a 1850 bp PCR product amplified with Atrx ex17 For and Rev primers. **(g)** Western blot analysis showing a loss of ATRX protein (275 KDa) (right panel). The faint truncated ATRX isoform (190 kDa) was also shown. * indicates positive H3.3^{G34R} *Atrx*$^{-/Y}$; *Tp53*$^{-/-}$ clones. (**h**) Generation of H3.3^{G34R} APT-tKO cells. Deletion of *Tert* exon 2 using *Tert* RNA guide #1 and #2 in H3.3^{G34R} *Atrx*$^{-/Y}$; *Tp53*$^{-/-}$ cells led to a truncation of a 1048 bp PCR product amplified with Tert ex2 g2F and g1R primers. * indicates positive H3.3^{G34R} APT-tKO clones. Source data are provided with this paper.

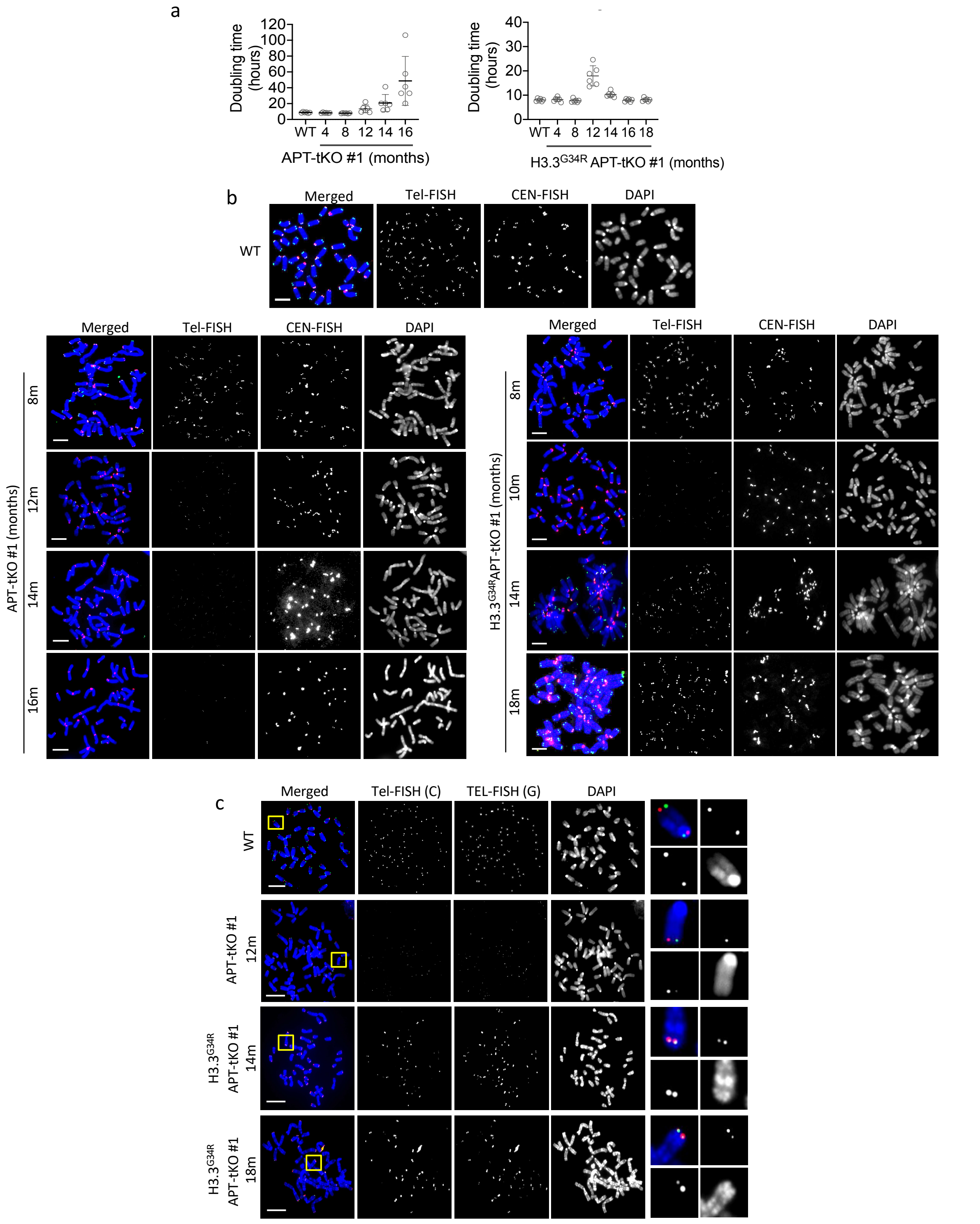

Supplementary Fig 2. Analyses of APT-tKO #1 and H3.3^{G34R}APT-tKO #1 cells.

(a) Changes in cell population doubling times in APT-tKO #1 and H3.3^{G34R}APT-tKO #1 cells over 18 months (n=6 independent experiments). Error bars are presented as mean values +/- SD. **(b)** DNA FISH analyses of APT-tKO #1 (left) and H3.3^{G34R}APT-tKO #1 (right) cells using telomeric TTAGGG (green) and minor satellite centromere (red) probes. **(c)** CO-FISH analyses showing examples of TSCE in H3.3^{G34R}APT-tKO #1 cells using telomeric CCCTAA (TEL-FISH (C); green) and TTAGGG (TEL-FISH (G); red) probes. **(b, c)** Scale bars: 5 μm; representative images from at least n=3 independent experiments. Source data are provided with this paper.

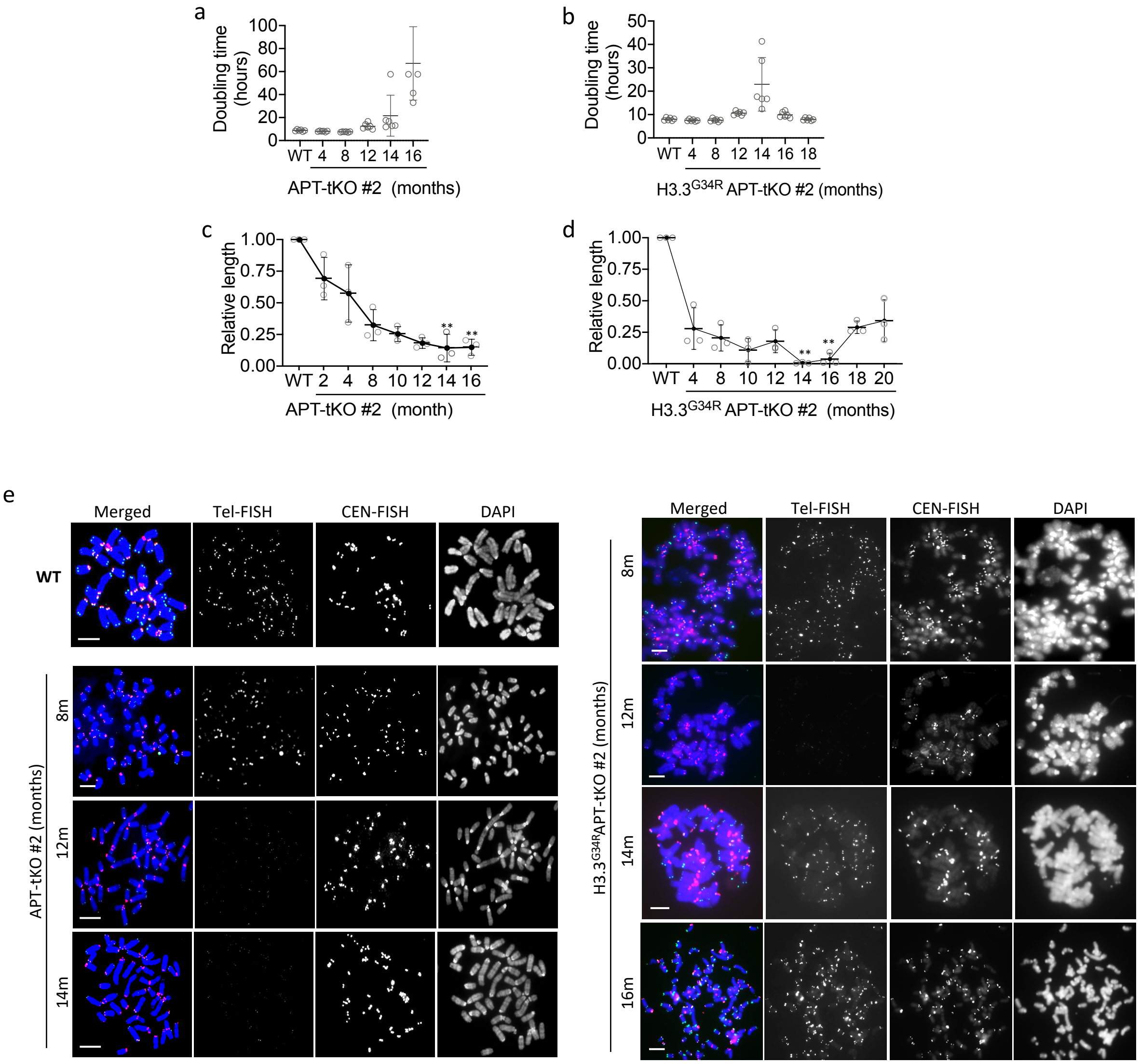

Supplementary Fig 3. Analyses of APT-tKO #2 and H3.3^{G34R}APT-tKO #2 cells.

(a, b) Changes in cell population doubling times in H3.3^{G34R}APT-tKO and APT-tKO cells over 18 months (n=6 independent experiments). **(c, d)** qPCR analyses of telomere length using primers against telomeric DNA and 36B4 control gene in APT-tKO #2 (c) and H3.3^{G34R}APT-tKO #2 (d) cells over a period of 20 months. n=3 experiments. **(e)** DNA FISH analyses of APT-tKO #2 (left) and H3.3^{G34R}APT-tKO #2 (right) cells using telomeric TTAGGG (green) and minor satellite centromere (red) probes. Scale bars: 5 µm; representative images from at least n=3 independent experiments. (a, b, c, d) Error bars are presented as mean values +/- SD. ** indicates *p* <0.005, Student's t-Test with two-tailed distribution. Source data are provided with this paper.

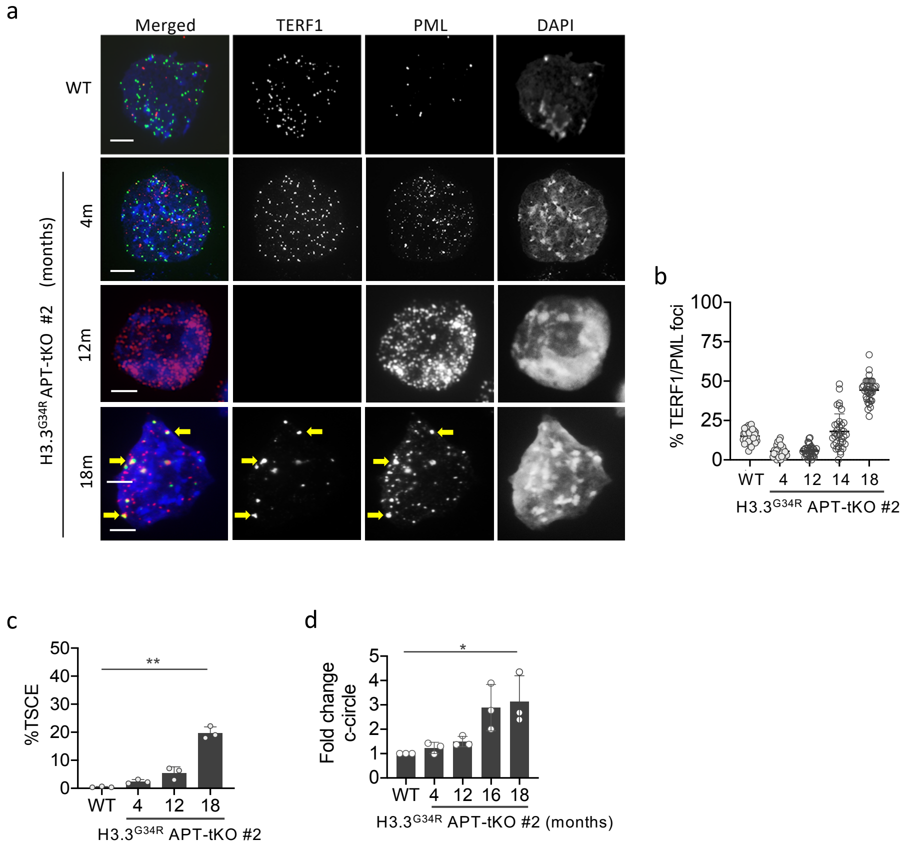

Supplementary Fig 4. Levels of APB formation and TSCE in H3.3^{G34R}APT-tKO cells.

(a) Immunostaining of APBs in H3.3^{G34R}APT-tKO #2 cells using antibodies against TERF1 (green; diluted at 1/500) and PML (red; diluted at 1/800)**.** Scale bars: 5 µm; representative images from at least n=3 independent experiments. **(b)** Percentages of co-localised TERF1 and PML foci are determined as percentages of telomeric/TERF1 foci that co-stained with PML over the total number of telomeric foci count (n=3 independent experiments; >1200 foci counted for each line). **(c)** Levels of TSCE in H3.3^{G34R}APT-tKO #2 cells were determined by CO-FISH analyses using telomeric CCCTAA (TEL-FISH (C)) and TTAGGG (TEL-FISH (G);) probes. Percentages of TSCE (% of telomeric ends) are shown (n=3 independent experiments, >1200 foci counted). **(d)** Fold increase of c-circle in H3.3^{G34R}APT-tKO #2 cells in comparison to WT cells (n=3 experiments). Error bars are presented as mean values +/- SD. ** indicates *p* <0.005 and * *p* < 0.05 Student's t-Test with two-tailed distribution. Source data are provided with this paper.

a

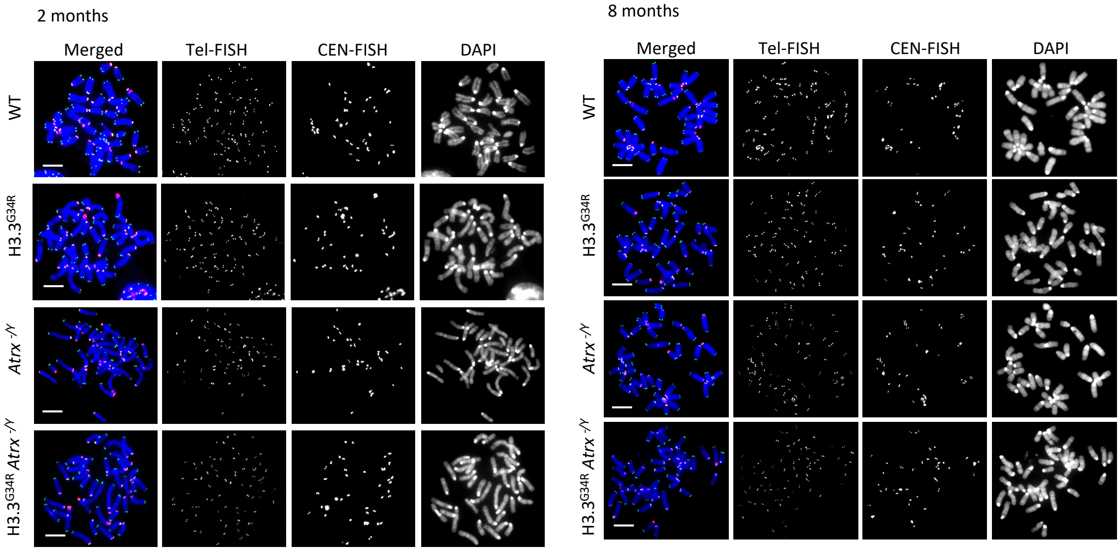

b

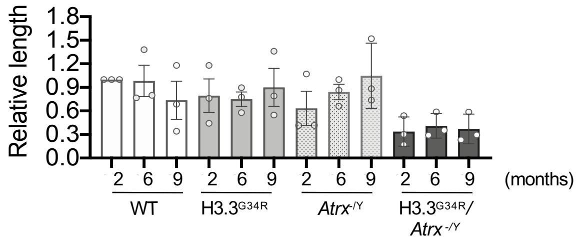

Supplementary Fig 5. Telomere FISH analyses of H3.3^{G34R}, *Atrx$^{-/Y}$*, and H3.3^{G34R} *Atrx$^{-/Y}$* cells.

(a) DNA FISH analyses of H3.3^{G34R}, *Atrx$^{-/-}$* and H3.3^{G34R} *Atrx $^{-/-}$* cells using telomeric TTAGGG (green) and minor satellite centromere (red) probes. Scale bars: 5 µm; representative images from at least n=3 independent experiments. **(b)** qPCR analyses of telomere length in H3.3^{G34R}, *Atrx$^{-/Y}$* and H3.3^{G34R}*Atrx$^{-/Y}$* cells over a period of 9 months using primers against telomeric DNA and 36B4 control gene (n=3 independent experiments). Error bars are presented as mean values +/- SD. Source data are provided with this paper.

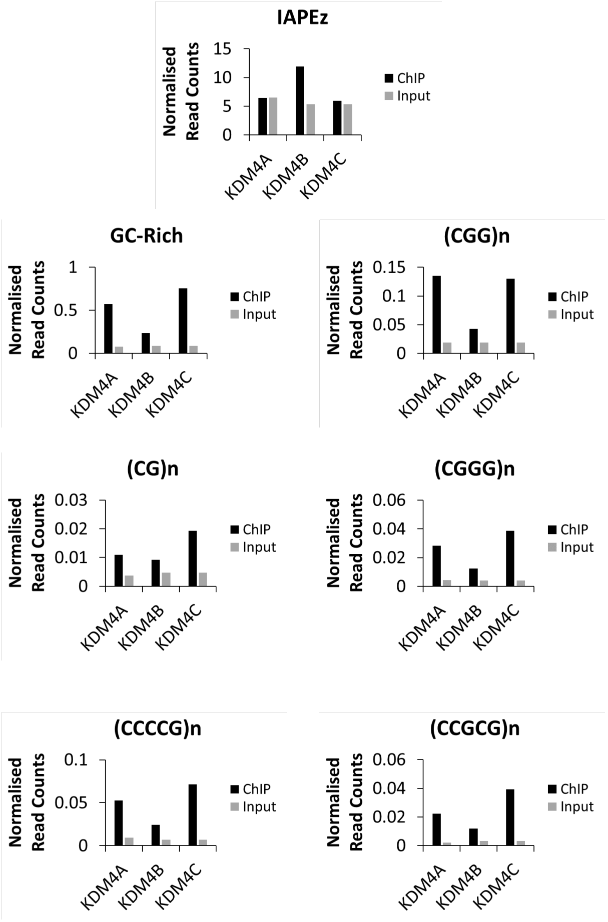

Supplementary Fig 6. ChIP-sequencing analysis of KDM4-A, -B and –C in mouse ES cells.
ChIP-sequencing analysis of KDM4-A, -B, and -C with input sequencing. Data shows reads which aligned to DNA repeats after normalising for total read counts.

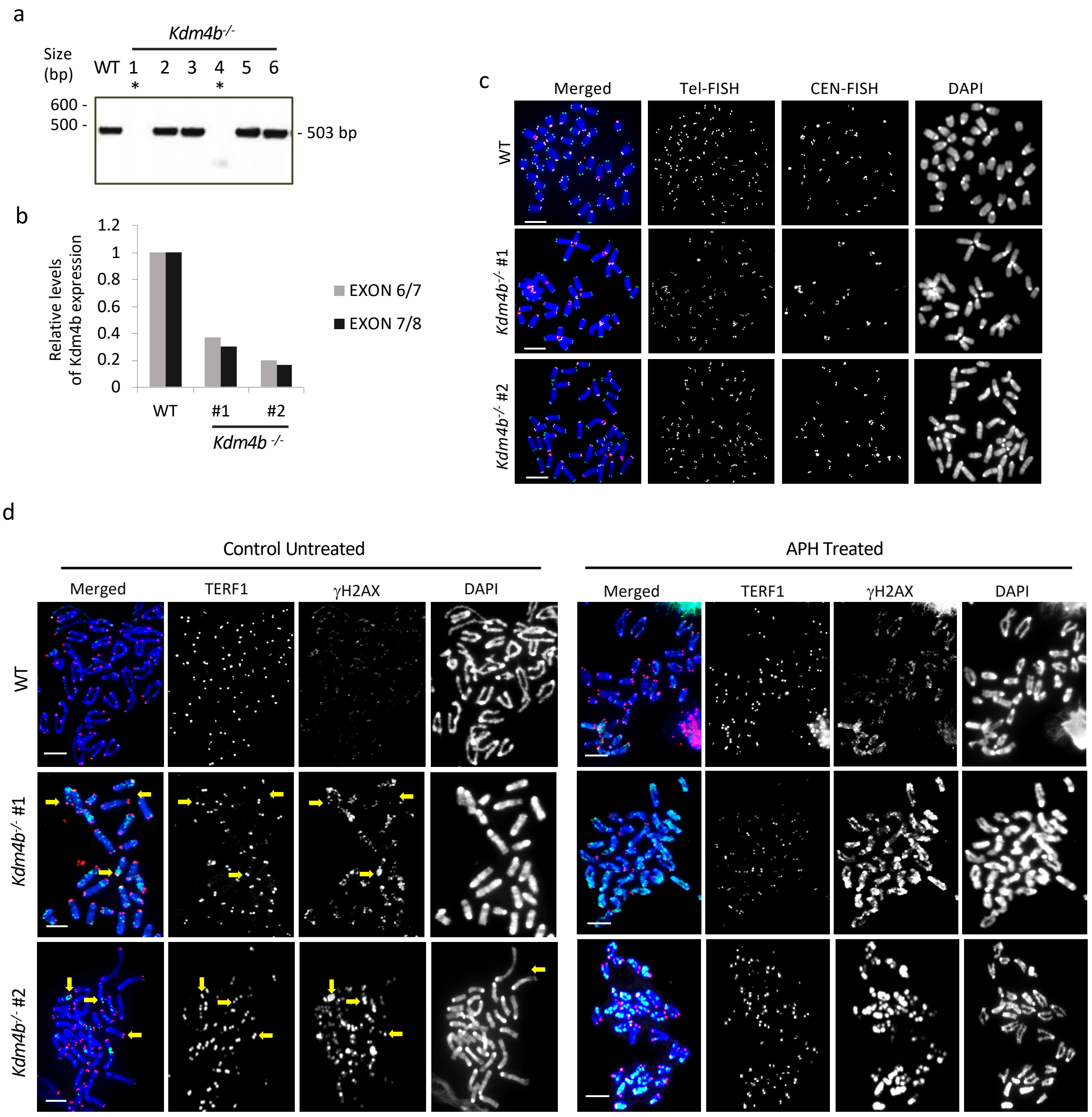

Supplementary Fig 7. *Kdm4b*$^{-/-}$ mouse ES cells show telomere replication stress phenotype.

(a) Generation of *Kdm4b*$^{-/-}$ cells. CRISPR-Cas9–mediated DNA editing system was used to knock out the *Kdm4b* gene in mouse ES cells. Targeted deletion of *Kdm4b* exons 3 to 5 using *Kdm4b* RNA guides #1 and #2 led to a loss of a 500 bp PCR product amplified with Kdm4b ex 3F and ex 3R primers. * indicates positive *Kdm4b*$^{-/-}$ clones. **(b)** Real time RT-PCR analysis showing reduction of *Kdm4b* RNA transcripts in *Kdm4b*$^{-/-}$ clones. Primers used were Kdm4b ex6/7 For and Rev, and Kdm4b ex7/8 For and Rev. **(c)** DNA FISH analyses of *Kdm4b*$^{-/-}$ #1 and #2 cells (3 months after *Kdm4b* knockout) using telomeric TTAGGG (green) and minor satellite centromere (red) probes. **(d)** Immunostaining of TERF1 (red, diluted in 1/500) and γH2AX (green; diluted at 1/800) in WT and *Kdm4b*$^{-/-}$ cells (clones #1 and #2) with and without 1mM APH treatment for 5 hours. Arrows indicate the presence of γH2AX at the telomere. **(c, d)** Scale bars: 5 µm; representative images from at least n=3 independent experiments. Source data are provided with this paper.

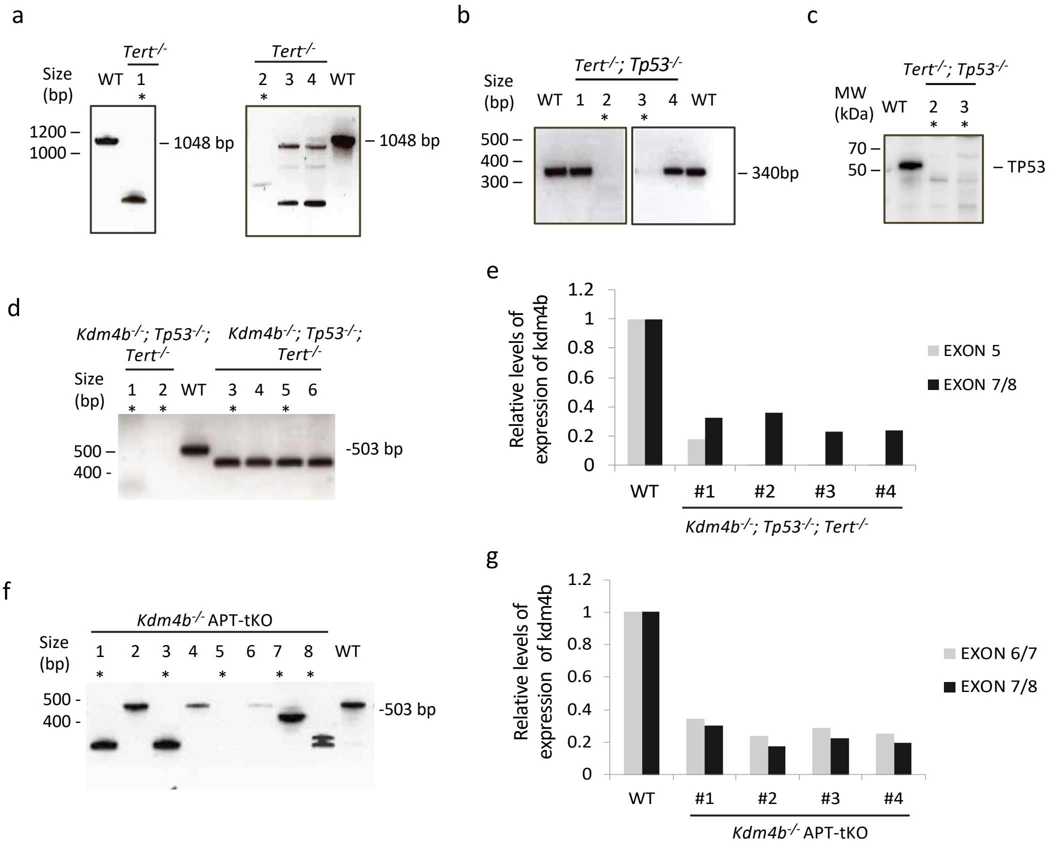

Supplementary Fig 8. Generation of *Kdm4b$^{-/-}$; Tp53$^{-/-}$; Tert$^{-/-}$* and *Kdm4b$^{-/-}$* APT-tKO mouse ES cells.

(a, b, c) CRISPR-Cas9–mediated DNA editing system was used to knock out the *Tert* gene in mouse ES cells **(a)**, followed by knockout of the *Tp53* gene. Targeted deletion of *Tert* exon 2 using *Tert* RNA guide #1 and #2 led to a reduction of a 1048 bp PCR product amplified with Tert ex2 g2F and g1R primers **(a).** Deletion of *Tp53* exon 4 in *Tert$^{-/-}$* cells using *Tp53* RNA guide #1 and #2 resulted in a reduction of a 340 bp PCR product amplified with Tp53 ex4 For and Rev primers **(b).** Western blot analyses showed loss of TP53 protein **(c). (d, f)** CRISPR-Cas9–mediated knockout of the *Kdm4b* gene in *TP53$^{-/-}$; TERT$^{-/-}$* and APT-tKO mouse ES cell lines that had been in culture for 10 months. Targeted deletion of *Kdm4b* exons 3 to 5 using *Kdm4b* RNA guides #1 and #2 led to a loss of a 503 bp PCR product amplified using Kdm4b ex 3F and ex 3R primers. * indicates positive *Kdm4b$^{-/-}$* clones. (**e, g)** Real time RT-PCR analysis showing reduction of *Kdm4b* RNA transcripts in positive *Kdm4b$^{-/-}$*; *Tp53$^{-/-}$; Tert$^{-/-}$* and *Kdm4b$^{-/-}$*APT-tKO ES cell lines. Primers used were Kdm4b ex5 For and Rev, Kdm4b ex6/7 For and Rev, and Kdm4b ex7/8 For and Rev, respectively. Source data are provided with this paper.

a

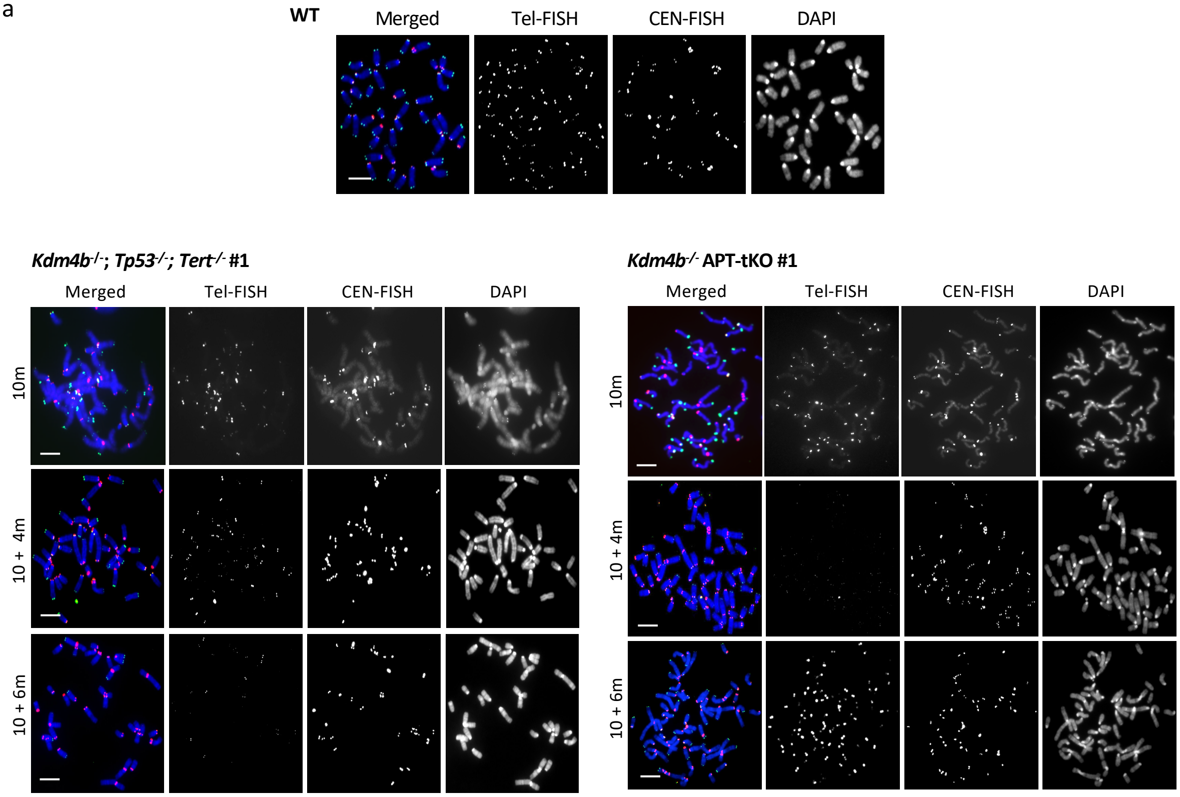

b

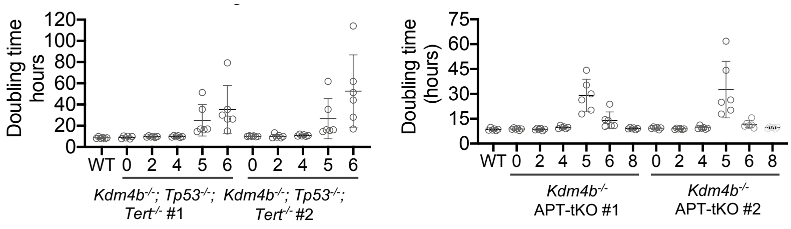

Supplementary Fig 9. Telomere length in *Kdm4b$^{-/-}$*; *Tp53$^{-/-}$; Tert$^{-/-}$ #1* and *Kdm4b$^{-/-}$*APT-tKO cells, and induction of ALT pathway in *Kdm4b$^{-/-}$* APT-tKO cells.

Kdm4b$^{-/-}$; *Tp53$^{-/-}$; Tert$^{-/-}$* and *Kdm4b$^{-/-}$*APT-tKO cell lines were created by knocking out the *Kdm4b* gene in *Tp53$^{-/-}$; Tert$^{-/-}$* and APT-tKO cell lines that had been cultured for 10 months. **(a)** DNA FISH analyses of *Kdm4b$^{-/-}$*; *Tp53$^{-/-}$; Tert$^{-/-}$ #1* and *Kdm4b$^{-/-}$* APT-tKO #1 cells using telomeric TTAGGG (green) and minor satellite centromere (red) probes. Scale bars: 5 µm; representative images from at least n=3 independent experiments. (**b**) Changes in cell population doubling times in *Kdm4b$^{-/-}$*; *Tp53$^{-/-}$; Tert$^{-/-}$* and *Kdm4b$^{-/-}$*APT-tKO cells over a period of 8 months following knockout of *Kdm4b$^{-/-}$* gene (n=6 independent experiments). Error bars are presented as mean values +/- SD. Source data are provided with this paper.

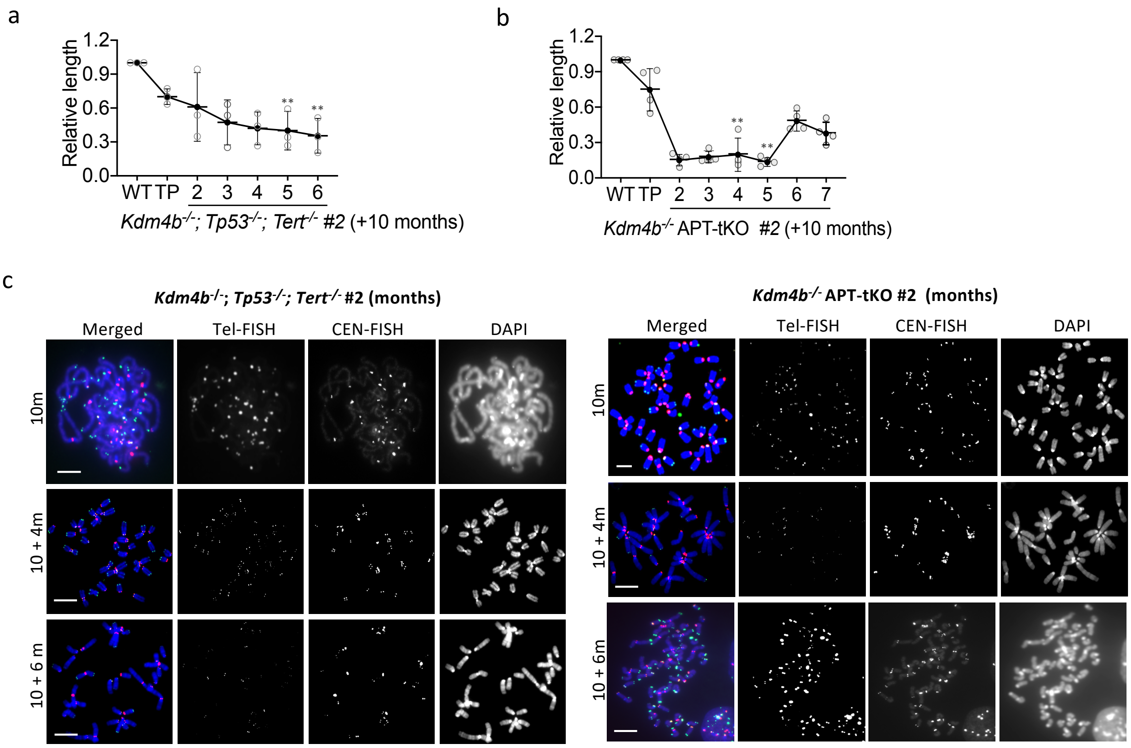

Supplementary Fig 10. Telomere length in *Kdm4b*$^{-/-}$; *Tp53*$^{-/-}$; *Tert*$^{-/-}$ #2 and *Kdm4b*$^{-/-}$APT-tKO #2 cells, and induction of ALT pathway in *Kdm4b*$^{-/-}$ APT-tKO #2 cells.

Kdm4b$^{-/-}$; *Tp53*$^{-/-}$; *Tert*$^{-/-}$ *#2* and *Kdm4b*$^{-/-}$APT-tKO #2 cell lines were created by knocking out the *Kdm4b* gene in *Tp53*$^{-/-}$; *Tert*$^{-/-}$ and APT-tKO cell lines that had been cultured for 10 month. **(a, b)** Telomere length analyses by qPCR using primers against telomeric DNA and 36B4 control gene in *Kdm4b*$^{-/-}$; *Tp53*$^{-/-}$; *Tert*$^{-/-}$ #2 (a; n=3 independent experiments) and *Kdm4b*$^{-/-}$ APT-tKO #2 (b; n=4 independent experiments) cells following the knockout of *Kdm4b* in *Tp53*$^{-/-}$; *Tert*$^{-/-}$ and APT-tKO cells. Error bars are presented as mean values +/- SD. ** indicates p <0.005 Student's t-Test with two-tailed distribution. Source data are provided with this paper. **(c)** DNA FISH analyses of *Kdm4b*$^{-/-}$; *Tp53*$^{-/-}$; *Tert*$^{-/-}$ *#2* and *Kdm4b*$^{-/-}$ APT-tKO #2 cells using telomeric TTAGGG (green) and minor satellite centromere (red) probes. Scale bars: 5 µm; representative images from at least n=3 independent experiments. Source data are provided with this paper.

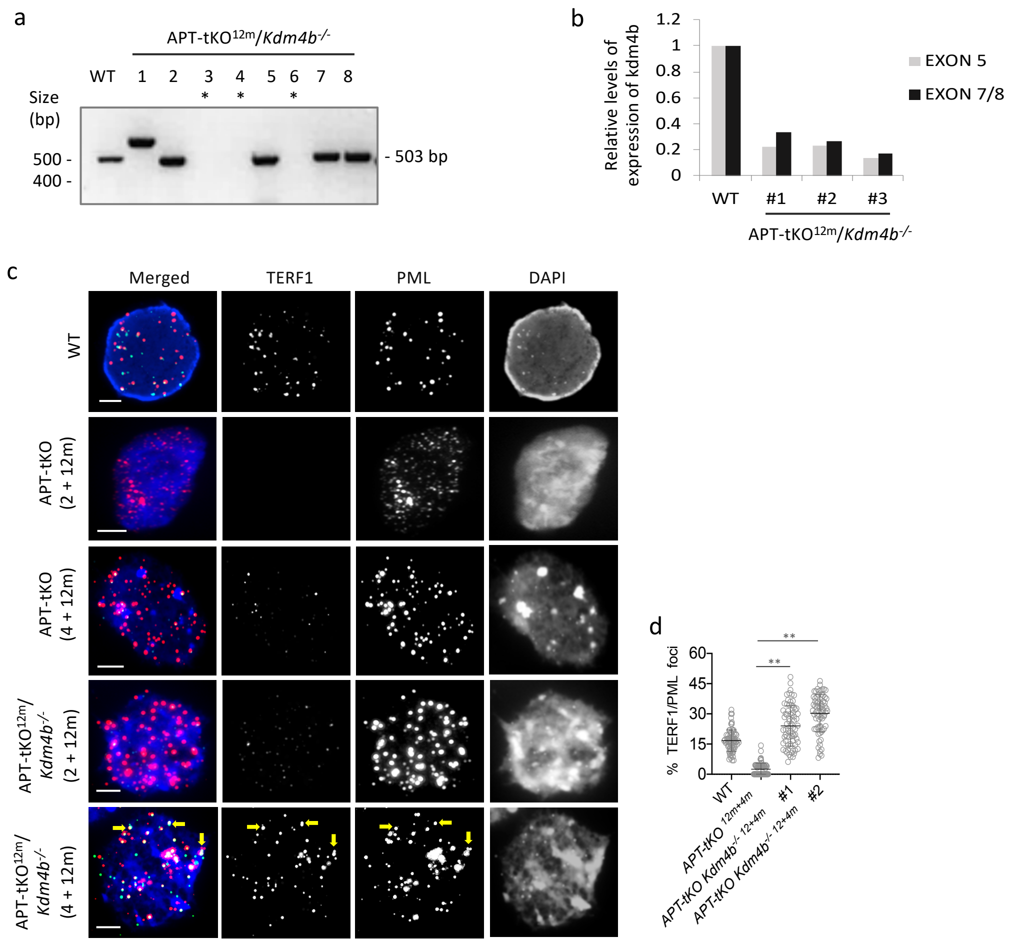

Supplementary Fig 11. Knockout of *Kdm4b* in APT-tKO^{12m} cells, and induction of ALT pathway in APT-tKO^{12m}/*Kdm4b*$^{-/-}$ cells .

(a) Knockout *Kdm4b* gene in APT-tKO^{12m} mouse ES cells. CRISPR-Cas9–mediated DNA editing system was used to knock out the *Kdm4b* gene in APT-tKO (APT-tKO^{12m}) cells that had been cultured for 12 months. Targeted deletion of *Kdm4b* exons 3 to 5 using *Kdm4b* RNA guides #1 and #2 led to a loss of a 503 bp PCR product amplified using Kdm4b ex 3F and ex 3R primers. * indicates positive APT-tKO^{12m}/*Kdm4b*$^{-/-}$ clones**.** **(b)** RT-PCR analyses showing reduction of *Kdm4b* RNA transcripts in APT-tKO^{12m}/*Kdm4b*$^{-/-}$ cells that were knocked out of the *Kdm4b* gene. **(c)** Immunostaining of TERF1 (green; diluted at 1/500) and PML (red; diluted at 1/800) in WT, APT-tKO^{12m} and APT-tKO^{12m}/*Kdm4b*$^{-/-}$ cells (2 to 4 months in culture following the knockout of *Kdm4b* gene). Arrows indicate the co-localised signals of TERF1 and APBs. Scale bars: 5 µm; representative images from at least n=3 independent experiments. **(d)** Percentages of co-localised TERF1 and PML foci are determined as percentages of telomeric/TERF1 foci that co-stained with PML over the total number of telomeric foci counted. (n=3 independent experiments ; >1500 telomeric foci counted for each line). Error bars are presented as mean values +/- SD. ** indicates p <0.005 Student's t-Test with two-tailed distribution. Source data are provided with this paper.

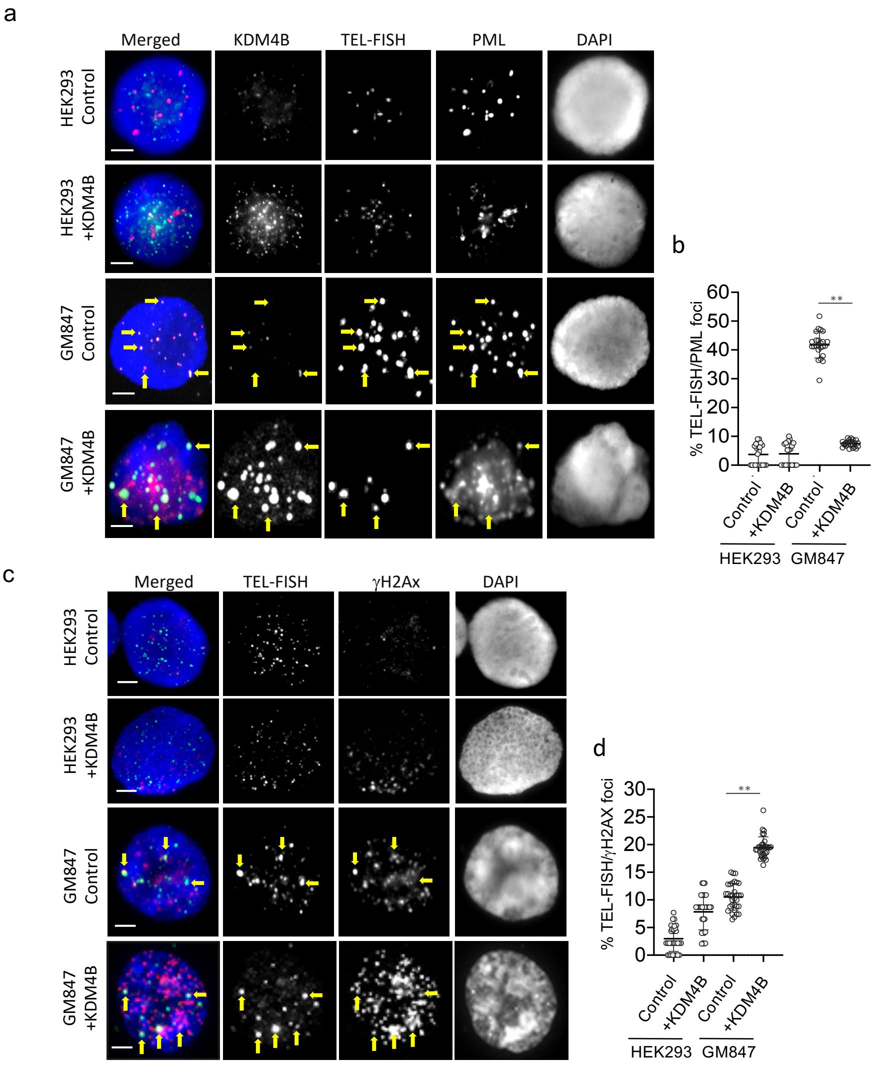

Supplementary Fig 12. KDM4B activity inhibits the ALT pathway.

(a) Immunostaining of KDM4B (green; diluted at 1/400), TEL-FISH (cyan), PML (red; diluted at 1/800) in control or KDM4B transfected HEK293 and GM847 cells. Arrows show KDM4B at telomeres. **(b)** Percentages of co-localized TEL-FISH and PML signals are determined as percentages of telomeric foci that co-stained with PML over the total number of telomeric foci counted (n=4 independent experiments, >1500 foci counted for each sample). **(c)** Immunostaining of γH2AX (red; diluted at 1/1500) and TEL-FISH (green) analyses in HEK293 and GM847 cells with and without overexpression of KDM4B (+KDM4B). Arrows show γ-H2AX at telomeres. **(d)** Percentages of co-localized TEL-FISH and γH2AX foci are determined as percentages of telomeric foci that co-stained with γH2AX over the total number of telomeric foci counted (n=4 independent experiments, >1500 foci counted for each line). **(a, c)** Scale bars: 5 µm; representative images from at least n=3 independent experiments. **(b, d)** Error bars are presented as mean values +/- SD. ** indicates *p* <0.005 Student's t-Test with two-tailed distribution. Source data are provided with this paper.

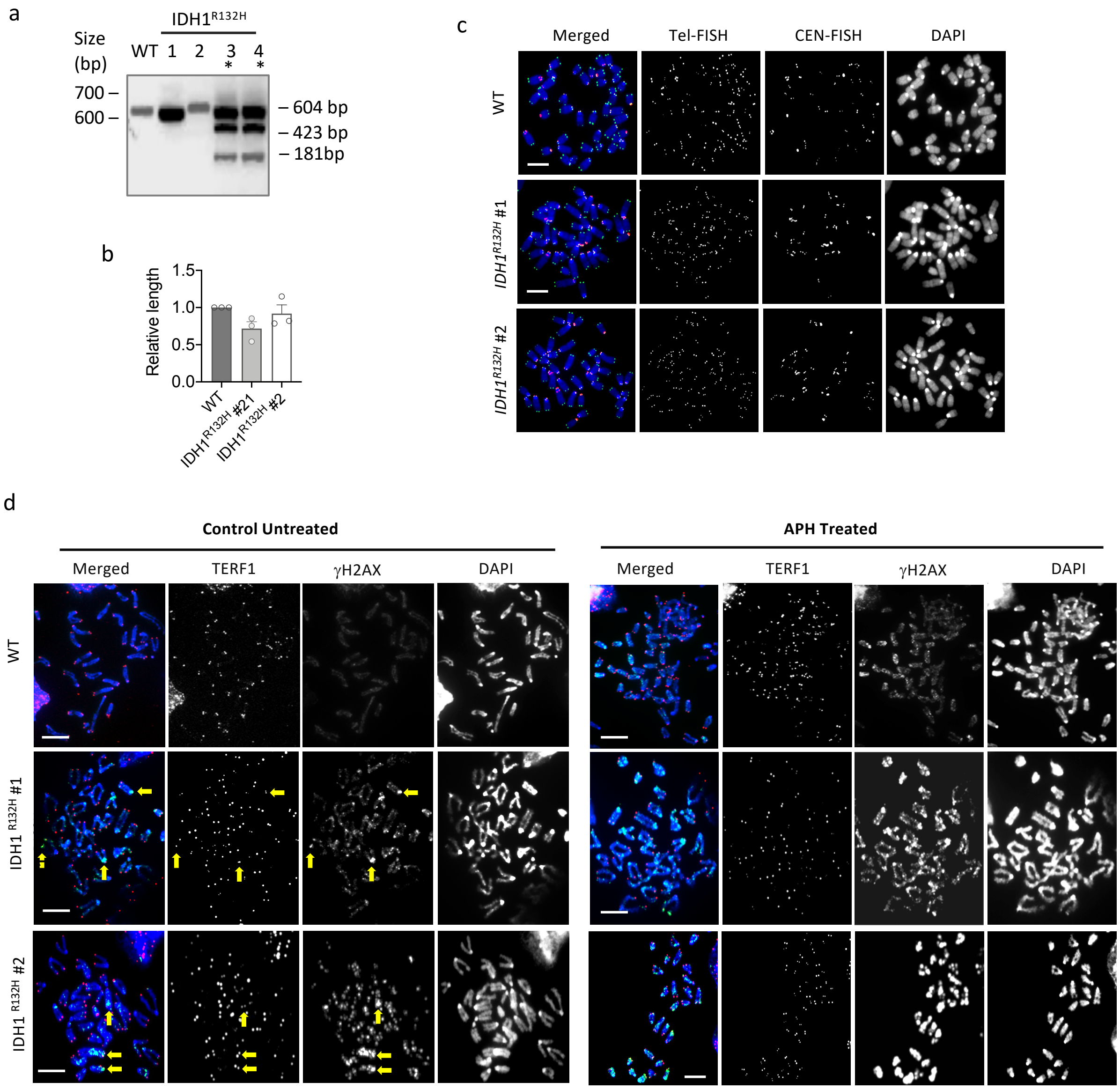

Supplementary Fig 13. Generation of IDH1^{R132H} mouse ES cells and IDH1^{R132H} cells show telomere replication stress phenotype.

(a) Generation of IDH1^{R132H} cells. CRISPR-Cas9–mediated DNA editing system was used to substitute the Arginine 132 residue of IDH1 with a Histidine (R132H) in mouse ES cells. Targeted mutation of *Idh1* exon 3 was achieved by transfecting *Idh1* RNA guide #1 and a DNA repair template carrying a R132H substitution. Positive clones were identified by restriction digest of a 604 bp PCR product amplified with Idh1 exon 3 For and Rev primers. * indicates positive IDH1^{R132H} clones. **(b)** Telomere length analyses of IDH1^{R132H} cells by qPCR using primers against telomeric DNA and 36B4 control gene (n=3 independent experiments). Error bars are presented as mean values +/- SD. **(c)** DNA FISH analyses of *Idh1$^{-/-}$* #1 and #2 cells using telomeric TTAGGG (green) and minor satellite centromere (red) probes. Scale bars: 5 µm. **(d)** Immunostaining of TERF1 (red) and γH2AX (green) in WT and IDH1^{R132H} cell lines (clones #1 and #2) with and without 1mM APH treatment for 5 hours. Arrows indicate the presence of γH2AX at the telomere. **(c, d)** Scale bars: 5 µm; representative images from at least n=3 independent experiments. Source data are provided with this paper.

Supplementary Table 1

Study ID	Sample ID	Patient ID	Altered	ATRX	TP53	H3F3A	IDH1
pediatric_dkfz_2017	ICGC_GBM38	ICGC_GBM38	1	MUTATED	G245S	no alteration	no alteration
pediatric_dkfz_2017	SJHGG041	SJHGG041	1	I360Rfs*6	H179D	G35R	no alteration
pediatric_dkfz_2017	ICGC_GBM43	ICGC_GBM43	1	K780Efs*12	R196*, R209Kfs*6	G35R	no alteration
pediatric_dkfz_2017	SJHGG100	SJHGG100	1	R188*	R248Q	K28M	no alteration
pediatric_dkfz_2017	ICGC_GBM15	ICGC_GBM15	1	R2131*	R273C	no alteration	R132H
pediatric_dkfz_2017	ICGC_GBM67	ICGC_GBM67	1	T887Rfs*19	R273C, T125M	no alteration	R132H
pediatric_dkfz_2017	ICGC_GBM5	ICGC_GBM5	1	K1045*	R342*	G35R	no alteration
pediatric_dkfz_2017	ICGC_GBM63	ICGC_GBM63	1	L764Vfs*2	R342*, R156Pfs*15	G35R	no alteration
pediatric_dkfz_2017	ICGC_GBM22	ICGC_GBM22	1	R1739*	T256Nfs*89	G35R	no alteration
pediatric_dkfz_2017	ICGC_GBM12	ICGC_GBM12	1	K1357Dfs*4	Y220C, T256P	K28M	no alteration
pediatric_dkfz_2017	SJHGG089	SJHGG089	1	X1817_splice	no alteration	no alteration	no alteration
pediatric_dkfz_2017	ICGC_GBM19	ICGC_GBM19	1	X1374_splice	no alteration	no alteration	no alteration
pediatric_dkfz_2017	ICGC_GBM50	ICGC_GBM50	1	X1374_splice	no alteration	no alteration	no alteration

Study ID	Sample ID	Patient ID	Altered	ATRX	TP53	H3F3A	IDH1	IDH2
glioma_mskcc_2019	P-0006182-T01-IM5	glioma_mskcc_2019_181	1	I360Rfs*6	R158G	G35R	no alteration	no alteration
glioma_mskcc_2019	P-0001731-T02-IM5	glioma_mskcc_2019_231	1	F888Sfs*17	R175H, R342*	G35V	no alteration	no alteration
glioma_mskcc_2019	P-0011213-T01-IM5	glioma_mskcc_2019_278	1	L513*	R273C	G35R	no alteration	no alteration
glioma_mskcc_2019	P-0002833-T01-IM3	glioma_mskcc_2019_297	1	K2182Vfs*8	H179Y	not profiled	no alteration	no alteration
glioma_mskcc_2019	P-0001471-T01-IM3	glioma_mskcc_2019_333	1	K1045*	I255del	not profiled	no alteration	no alteration
glioma_mskcc_2019	P-0001730-T01-IM3	glioma_mskcc_2019_416	1	R840Kfs*9	no alteration	not profiled	R132H	no alteration
glioma_mskcc_2019	P-0009629-T01-IM5	glioma_mskcc_2019_418	1	R781*	V157F	no alteration	R132G	no alteration
glioma_mskcc_2019	P-0006546-T01-IM5	glioma_mskcc_2019_420	1	L2258Gfs*10	H193R	no alteration	R132H	no alteration
glioma_mskcc_2019	P-0009216-T01-IM5	glioma_mskcc_2019_425	1	K319*	R249T	no alteration	R132H	no alteration
glioma_mskcc_2019	P-0013676-T01-IM5	glioma_mskcc_2019_44	1	L1746*, G51V	R196*, G245S	P44H	no alteration	R353H, D312N, A174T
glioma_mskcc_2019	P-0013602-T01-IM5	glioma_mskcc_2019_743	1	L287*	R282G	no alteration	R132H	no alteration
glioma_mskcc_2019	P-0015289-T01-IM6	glioma_mskcc_2019_751	1	X1519_splice	H179R	no alteration	R132H	no alteration
glioma_mskcc_2019	P-0016048-T01-IM6	glioma_mskcc_2019_778	1	L359Tfs*3	R342P	K28M	no alteration	no alteration
glioma_mskcc_2019	P-0006545-T01-IM5	glioma_mskcc_2019_92	1	E1664*	C238Y, L137Wfs*33	G35R	no alteration	no alteration
glioma_mskcc_2019	P-0006410-T01-IM5	glioma_mskcc_2019_99	1	F2113Sfs*9	R342*	G35V	no alteration	no alteration

Supplementary Table 2. Guide RNA and primer sequences

**CRISPR-Cas9 targeting**						
Genes	Guide	Targeting Exon	guide RNA	Vector	Primers for PCR screen	Primer sequences
Atrx	# 1	17	CACCGCTTCGGCAGCCATCCCAAGC	CRISPR-Cas9 px330 (Addgene)	*Atrx ex17 For*	CAGTGTTAAGAGGACAAACC
	# 2	17	AAACGCTTGGGATGGCTGCCGAAGC	CRISPR-Cas9 px330 (Addgene)	*Atrx ex17 Rev*	GATGCTTCACCTACTAAGTGTC
Tp53	# 1	4	AGTGAAGCCCTCCGAGTGTC	pGS-gRNA-Neo (Genscript)	*Tp53 ex4 For*	CAGTCCTGAGGGTTCTTCTTTG
	# 2	4	AACAGATCGTCCATGCAGTG	pGS-gRNA-Neo (Genscript)	*Tp53 ex4 Rev*	GCATTGAAAGGTCACACGAAAG
Tert	# 1	2	GCCAGTATCGACGCGATAGA	pLentiCrispr V2 (Genscript)	*Tert ex2 g2F*	AAGACCCTCTTCTCCTTACCA
	# 2	2	GCGCAACGAGAGAAACGTGC	pLentiCrispr V2 (Genscript)	*Tert ex2 g1R*	CCTGAGGAGTCTGACATATTGG
	# 4	2	TACAGGCCCACCCGACCCGT	pLentiCrispr V2 (Genscript)	*Tert ex2 g4F*	TGCATGGATGCTACTGTTGAG
	# 5	2	GAAGCGGCGCTCATTGTGCC	pLentiCrispr V2 (Genscript)	*Tert ex2 g5R*	CCAACCACGGACTGCATAAA
Kdm4b	# 1	3	GTATGATGACATCGACGATG	CRISPR-Cas9 px330 (Addgene)	*Kdm4b ex 3F*	GGCGTGAATACACCCTACTT
	# 2	5	GCACAGGGAACTGAGCGTAT	CRISPR-Cas9 px330 (Addgene)	*Kdm4b ex 3R*	GCAGGTAGTTGATGCTGTAGA
Idh1	# 1	3	TCATTGGCCGACATGCATAT	pLentiCrispr V2 (Genscript)	*Idh1 ex3 For*	CAAGTTGAAACAAATGTGGAAATCC
					Idh1 ex3 Rev	GCTCTATGGTGGGACTCAAATAC

IDH1R132H DNA template

TAT GAT TTA GGC ATA GAG AAT CGT GAT GCC ACC AAT GAC CAG GTC ACC AAA GAT GCT GCA GAG GCT ATA AAG AAA TAC AAC GTG GGC GTC AAG TGT GCT ACC ATC ACC CCC GAT GAG AAG AGG GTT GAA GAA TTC AAG TTG AAA CAA ATG TGG AAA TCC CCA AAT GGC ACC ATC CGA AAC ATT CTG GGT GGC ACT GTC TTC AGG GAA GCT ATT ATC TGC AAA AAT ATC CCC CGG CTA GTG ACA GGC TGG GTA AAA CCC ATC *ATC ATT GGC **CAC** CAT GCA TAT **GGG*** GAC CAA GTA AGT CAT TTG AGT GAG CAT GAA TTC ATT TTT TCC GTA TTT ATT TTT CAC AGA CCT TTA GCT CCA ATA TCT ATG TTT GTT CTT TGG GCA TCT ACA CCC TTA AGA GAG GTC CAA CAT ATA TTA TGA ATA AAC TAT AGA TTT ACA TAT GTT TAG GAA AGT GTA AAA ATA CAG ATA AAC TTT CAA ATA AAT ACT CAT CTT AAC ACA TTT TCC CCA AAT ATC CTT TTG TTA AGT TAT TCC ATA TGA TAT TGC CAT TTT TGT ATT AGT AAG ACC ATA CGC TCA GAT CAG TGG TTT TCA

The substitutions of CGA to CAC (Arginine 132 to Histidine) and GGG to GGA (this change destroys the PAM site but does not change the codon) are highlighted as bold texts.
EcoRI sites are underlined, gRNA targeting site is italised

Primers for ChIP/qPCR and telomere length measurement

Telomere Forward	CGGTTTGTTTGGGTTTGGGTTTGGGTTTGGGTTTGGGTT

Telomere Reverse	GGCTTGCCTTACCCTTACCCTTACCCTTACCCTTACCCT
Gapdh pro For	AGAGAGGGAGGAGGGGAAATG
Gapdh pro Rev	AACAGGGAGGAGCAGAGAGCAC
Mouse 36B4 For	ACTGGTCTAGGACCCGAGAAG
Mouse 36B4 Rev	TCAATGGTGCCTCTGGAGATT
**Primers for RT-qPCR**	
Gapdh For	GTGGAGTCTACTGGTGTCTTC
Gapdh Rev	GGTTCACACCCATCACAAAC
Kdm4b ex5 For	GGCGTGAATACACCCTACTT
Kdm4b ex5 Rev	GCAGGTAGTTGATGCTGTAGA
Kdm4b ex6/7 For	CCTGGCCATAGGCTTCTTC
Kdm4b ex6/7 Rev	CGTACTTCTTCAGGATGATGGG
Kdm4b ex7/8 For	GCCTTCCTAAGGCACAAGAT
Kdm4b ex7/8 Rev	CCCAGCTTCCTGTGTAATCC
