# Supplementary material for: Mutations inhibiting KDM4B drive ALT activation in ATRX-mutated glioblastomas
Source: Nat Commun. 2021 May 10;12:2584. doi: 10.1038/s41467-021-22543-z (PMC8110556; doi:10.1038/s41467-021-22543-z)
Supplement: Supplementary file 2 — Reporting Summary [file 41467_2021_22543_MOESM2_ESM.pdf]

nature research

Corresponding author(s): Lee Wong and Joanna Voon

Last updated by author(s): 2021/2/19

Reporting Summary

Nature Research wishes to improve the reproducibility of the work that we publish. This form provides structure for consistency and transparency in reporting. For further information on Nature Research policies, see our Editorial Policies and the Editorial Policy Checklist.

Statistics

For all statistical analyses, confirm that the following items are present in the figure legend, table legend, main text, or Methods section.

n/a	Confirmed	
☐	☒	The exact sample size (n) for each experimental group/condition, given as a discrete number and unit of measurement
☐	☒	A statement on whether measurements were taken from distinct samples or whether the same sample was measured repeatedly
☐	☒	The statistical test(s) used AND whether they are one- or two-sided<br>*Only common tests should be described solely by name; describe more complex techniques in the Methods section.*
☐	☒	A description of all covariates tested
☐	☒	A description of any assumptions or corrections, such as tests of normality and adjustment for multiple comparisons
☐	☒	A full description of the statistical parameters including central tendency (e.g. means) or other basic estimates (e.g. regression coefficient) AND variation (e.g. standard deviation) or associated estimates of uncertainty (e.g. confidence intervals)
☐	☒	For null hypothesis testing, the test statistic (e.g. F, t, r) with confidence intervals, effect sizes, degrees of freedom and P value noted<br>*Give P values as exact values whenever suitable.*
☒	☐	For Bayesian analysis, information on the choice of priors and Markov chain Monte Carlo settings
☒	☐	For hierarchical and complex designs, identification of the appropriate level for tests and full reporting of outcomes
☒	☐	Estimates of effect sizes (e.g. Cohen's d, Pearson's r), indicating how they were calculated

Our web collection on statistics for biologists contains articles on many of the points above.

Software and code

Policy information about availability of computer code

Data collection	Zeiss Imager M2 Fluorescence microscope and Zen Imaging Software were used for immunofluorescence data collection.<br>Image Lab 5.2.1 Imaging System (Biorad) was used for Western blot image capture.<br>LightCycler ® 96 Software (Roche Life Science) was used for quantitative PCR analysis.
Data analysis	Microsoft Excel was used to analyse data.<br>Repeat Enrichment Estimator v1.062 and Repbase database were used for alignment of repeat sequences (see Day DS, Luquette LJ, Park PJ, Kharchenko PV. Estimating enrichment of repetitive elements from high-throughput sequence data. Genome Biol 11, R69 (2010))

For manuscripts utilizing custom algorithms or software that are central to the research but not yet described in published literature, software must be made available to editors and reviewers. We strongly encourage code deposition in a community repository (e.g. GitHub). See the Nature Research guidelines for submitting code & software for further information.

Data

Policy information about availability of data

All manuscripts must include a data availability statement. This statement should provide the following information, where applicable:

- Accession codes, unique identifiers, or web links for publicly available datasets
- A list of figures that have associated raw data
- A description of any restrictions on data availability

Source Data are available for Figures 1 to 5 and Suppl Figures 1 to 13

Field-specific reporting

Please select the one below that is the best fit for your research. If you are not sure, read the appropriate sections before making your selection.

☒ Life sciences ☐ Behavioural & social sciences ☐ Ecological, evolutionary & environmental sciences

For a reference copy of the document with all sections, see nature.com/documents/nr-reporting-summary-flat.pdf

Life sciences study design

All studies must disclose on these points even when the disclosure is negative.

Sample size	Yes, information on sample size was provided in Figure Legends and Source Data. Sample sizes were chosen based on previous publications in the field.
Data exclusions	No data was excluded.
Replication	Number of experiments are indicated in the Figure Legends. All experiments were conducted with at least n=3 biological replicates.
Randomization	Randomisation is not applicable, as samples were not assigned to experimental groups.
Blinding	Automated image analysis was used where appropriate to minimize bias. Experiments were performed by different researchers providing reproducible and statistical relevant results.

Reporting for specific materials, systems and methods

We require information from authors about some types of materials, experimental systems and methods used in many studies. Here, indicate whether each material, system or method listed is relevant to your study. If you are not sure if a list item applies to your research, read the appropriate section before selecting a response.

Materials & experimental systems

n/a	Involved in the study	
☐	☒	Antibodies
☐	☒	Eukaryotic cell lines
☒	☐	Palaeontology and archaeology
☒	☐	Animals and other organisms
☒	☐	Human research participants
☒	☐	Clinical data
☒	☐	Dual use research of concern

Methods

n/a	Involved in the study	
☒	☐	ChIP-seq
☒	☐	Flow cytometry
☒	☐	MRI-based neuroimaging

Antibodies

Antibodies used	Information provided in the Method and Material Section.<br>Antibodies used were directed against anti-H3 (Abcam, #ab1791), anti-H3.3 (Abcam, #ab176840), anti-H3K9me3 (Abcam, ab8898), anti-H3K36me3 (Abcam, #ab9050), anti-?H2A.X/phospho-histone H2A.X (Ser139) clone JBW301 (Merck Millipore, #05-636), anti-ATRX (Santa Cruz Biotechnologies, #sc15408), anti- KDM4B (Abcam, #ab191434) anti-IDH1 (Sigma Aldrich, SAB4100064), anti-IDH1R132H (Sigma Aldrich, SAB4200548), anti-HP1? (Merck Millipore, #MAB3584), anti-PML (Merck Millipore, #MAB3738), anti-TP53 (Cell Signaling Technologies, #cst-2524), anti-Flag (Sigma, #F1804), anti-TERF1 (Alpha Diagnostics, #TRF12-S) and anti-BrdU (Abcam, #ab6326), Anti-β Actin (AC-15) (Santa Cruz Biotechnology, #sc69879), Goat anti Rabbit IgG, HRP conjugate (Merck Millipore, #AP187P), Donkey anti-Mouse IgG, HRP conjugate (Merck Millipore, #AP192P), Donkey anti-Mouse IgG (H+L), Alexa Fluor 594 (Invitrogen, #A-21203), Donkey anti-Rabbit IgG (H+L), Alexa Fluor 488 (Invitrogen, # A-21206), Donkey anti-Mouse IgG (H+L), Alexa Fluor 488 (Invitrogen, #A-21202), Chicken anti-Rabbit IgG (H+L), Alexa Fluor 594 (Invitrogen, #A-21442)
Validation	Uncropped Western blots provided in Source Data. Additional information provided on the manufacturer's website.<br>anti-H3 (Abcam, #ab1791) was validated in ChIP, WB, IP, ICC (https://www.abcam.com/histone-h3-antibody-nuclear-marker-and-chip-grade-ab1791.html) by the manufacturer and has been used in 3267 publications.<br>anti-H3.3 (Abcam, #ab176840) was validated in ChIP-sequencing, ICC/IF, ChIP, IHC-P, WB, Dot blot (https://www.abcam.com/histone-h33-antibody-epr17899-chip-grade-ab176840.html) by the manufacturer and has been used in 9 publications.<br>anti-H3K9me3 (Abcam, ab8898) was validated in WB, IHC-P, ICC/IF, ChIP (https://www.abcam.com/histone-h3-tri-methyl-k9-antibody-chip-grade-ab8898.html) by the manufacturer and has been used in 1185 publications.<br>anti-H3K36me3 (Abcam, #ab9050) was validated in ICC/IF, WB, ChIP (https://www.abcam.com/histone-h3-tri-methyl-k36-antibody-chip-grade-ab9050.html) by the manufacturer and has been used in 711 publications.<br>anti-H2A.X/phospho-histone H2A.X (Ser139) clone JBW301 (Merck Millipore, #05-636) was validated in ICC, IF, WB, ChIP, IHC (https://www.merckmillipore.com/AU/en/product/Anti-phospho-Histone-H2A.X-Ser139-Antibody-clone-JBW301,MM_NF-05-636) by the manufacturer and has been used in 434 publications.

anti-ATRX (Santa Cruz Biotechnologies, #sc15408) – (https://www.scbt.com/p/atrx-antibody-h-300) has been used in 30 publications.
anti- KDM4B (Abcam, #ab191434) was validated in WB, IHC-P, ICC/IF, Flow Cytometry, IP (https://www.abcam.com/kdm4b--jmjd2b-antibody-epr18603-ab191434.html) by the manufacturer.
anti-IDH1 (Sigma Aldrich, SAB4100064) was validated in WB (https://www.sigmaaldrich.com/catalog/product/sigma/sab4100064?lang=en&region=AU) by the manufacturer and has been used in 5 publications.
anti-IDH1R132H (Sigma Aldrich, SAB4200548) was validated in WB https://www.sigmaaldrich.com/catalog/search?term=SAB4200548&interface=All&N=0&mode=match%20partialmax&lang=en&region=AU&focus=product) by the manufacturer and has been used in 8 publications.
anti-HP1[illegible] (Merck Millipore, #MAB3584) was validated in Elisa, ICC, IHC (https://www.merckmillipore.com/AU/en/product/Anti-Heterochromatin-Protein-1-Antibody-clone-2HP-1H5,MM_NF-MAB3584?ReferrerURL=https%3A%2F%2Fwww.google.com%2F&bd=1) by the manufacturer and has been used in 20 publications.
anti-PML (Merck Millipore, #MAB3738) was validated in ICC, IP, WB (https://www.merckmillipore.com/AU/en/product/Anti-PML-Antibody-clone-36.1-104,MM_NF-MAB3738) by the manufacturer and has been used in 12 publications.
anti-TP53 (Cell Signaling Technologies, #cst-2524) was validated in WB, IP, IF, ICH, Flow Cytometry, ChIP (https://www.cellsignal.com/products/primary-antibodies/p53-1c12-mouse-mab/2524) by the manufacturer and has been used in 663 publications.
anti-Flag (Sigma, #F1804) was validated in WB, IP, IHC, IF, ICC (https://www.sigmaaldrich.com/catalog/product/sigma/f1804?lang=en&region=AU&gclid=CjwKCAiAmrOBBhA0EiwArn3mfIjhI5lkTPYSicZO-ASu0XZ3fBnfznbKPjgGaw7pfAn9_-80KgbQrRoCZMMQAvD_BwE) by the manufacturer and has been used in 5273 publications.
anti-TERF1 (Alpha Diagnostics, #TRF12-S) was validated in IF, ICC (https://www.4adi.com/4adi/anti-mouse-telomeric-repeat-binding-factor-1-trf1-antiserum-2-13296-p.html) by the manufacturer and has been used in 7 publications.
anti-BrdU (Abcam, #ab6326) was validated in ICC/IF, IHC-P, Flow Cyt (https://www.abcam.com/brdu-antibody-bu175-icr1-proliferation-marker-ab6326.html) by the manufacturer and has been used in 1165 publications.
Anti-β Actin (AC-15) (Santa Cruz Biotechnology, #sc69879) was validated in WB, IP, IF (https://www.scbt.com/p/beta-actin-antibody-ac-15) by the manufacturer and has been used in 855 publications.
Goat anti Rabbit IgG, HRP conjugate (Merck Millipore, #AP187P) was validated in WB (https://www.merckmillipore.com/AU/en/product/Goat-Anti-Rabbit-IgG-Antibody-HRP-conjugate-Species-Adsorbed,MM_NF-AP187P?ReferrerURL=https%3A%2F%2Fwww.google.com%2F&bd=1) by the manufacturer and has been used in 2 publications.
Donkey anti-Mouse IgG, HRP conjugate (Merck Millipore, #AP192P) was validated in WB and Elisa (https://www.merckmillipore.com/AU/en/product/Donkey-Anti-Mouse-IgG-Antibody-HRP-conjugate-Species-Adsorbed,MM_NF-AP192P?ReferrerURL=https%3A%2F%2Fwww.google.com%2F) by the manufacturer and has been used in 17 publications.
Donkey anti-Mouse IgG (H+L), Alexa Fluor 594 (Invitrogen, #A-21203) was validated in IF, ICC (https://www.thermofisher.com/antibody/product/Donkey-anti-Mouse-IgG-H-L-Highly-Cross-Adsorbed-Secondary-Antibody-Polyclonal/A-21203) by the manufacturer and has been used in 83 publications.
Donkey anti-Rabbit IgG (H+L), Alexa Fluor 488 (Invitrogen, # A-21206) was validated in IF, ICC - (https://www.thermofisher.com/antibody/product/Donkey-anti-Rabbit-IgG-H-L-Highly-Cross-Adsorbed-Secondary-Antibody-Polyclonal/A-21206) by the manufacturer and has been used in 400 publications.
Alexa Fluor 488 (Invitrogen, #A-21202) was validated in IF, ICC (https://www.thermofisher.com/antibody/product/Donkey-anti-Mouse-IgG-H-L-Highly-Cross-Adsorbed-Secondary-Antibody-Polyclonal/A-21202) by the manufacturer and has been used in 307 publications.
Chicken anti-Rabbit IgG (H+L), Alexa Fluor 594 (Invitrogen, #A-21442) was validated in IF (https://www.thermofisher.com/antibody/product/Chicken-anti-Rabbit-IgG-H-L-Cross-Adsorbed-Secondary-Antibody-Polyclonal/A-21442) by the manufacturer and has been used in 32 publications.

Eukaryotic cell lines

Policy information about cell lines

Cell line source(s)	Information provided in the Method and Material Section.<br>B143, HEK293, GM847 and U2OS cells were obtained from the American Type Culture Collection.
Authentication	Cell lines were authenticated by 16-locus short-tandem-repeat profiling by CellBank Australia.
Mycoplasma contamination	Cell lines were tested for mycoplasma contamination by CellBank Australia.
Commonly misidentified lines<br>(See ICLAC register)	No commonly misidentified cell lines were used
