# Supplementary figures and images for: Mutations inhibiting KDM4B drive ALT activation in ATRX-mutated glioblastomas
Source: Nat Commun. 2021 May 10;12:2584. doi: 10.1038/s41467-021-22543-z (PMC8110556; doi:10.1038/s41467-021-22543-z)

2a

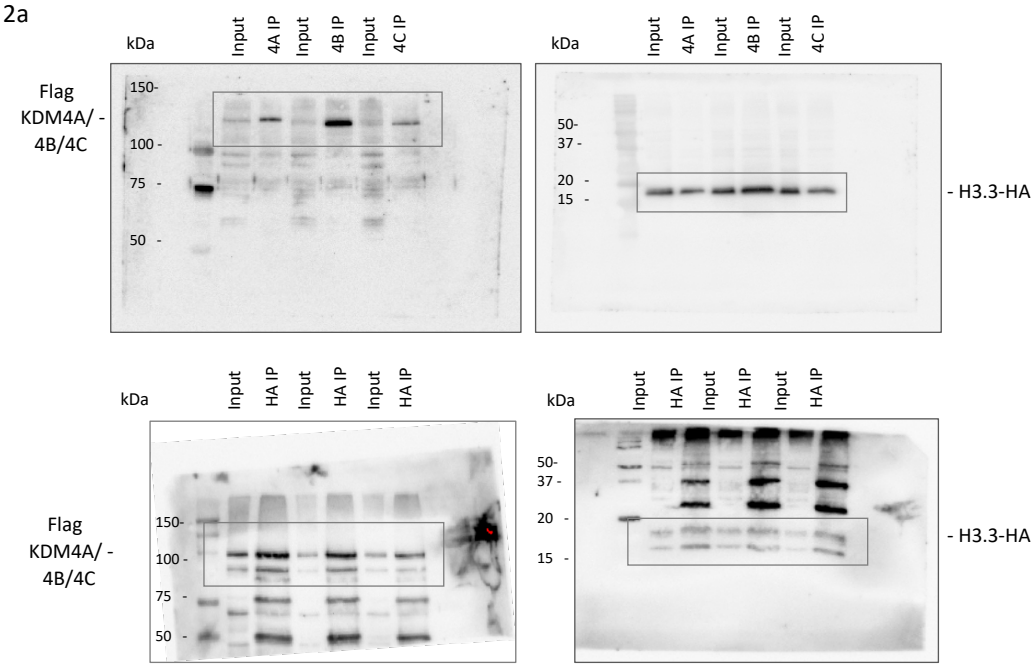

2d

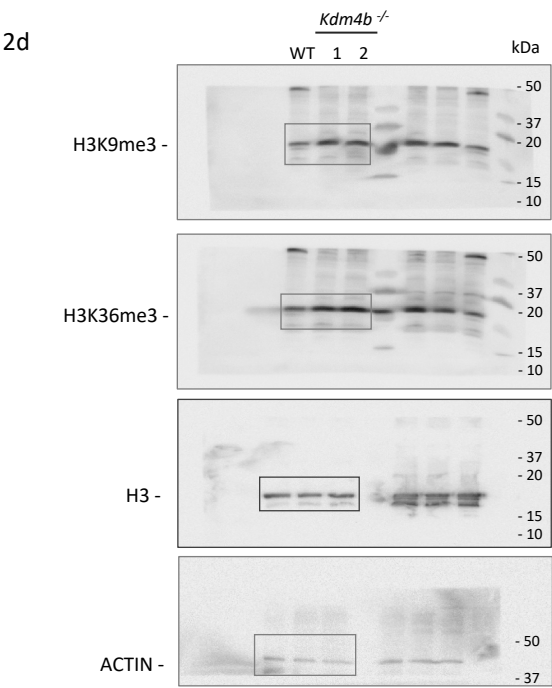

4c

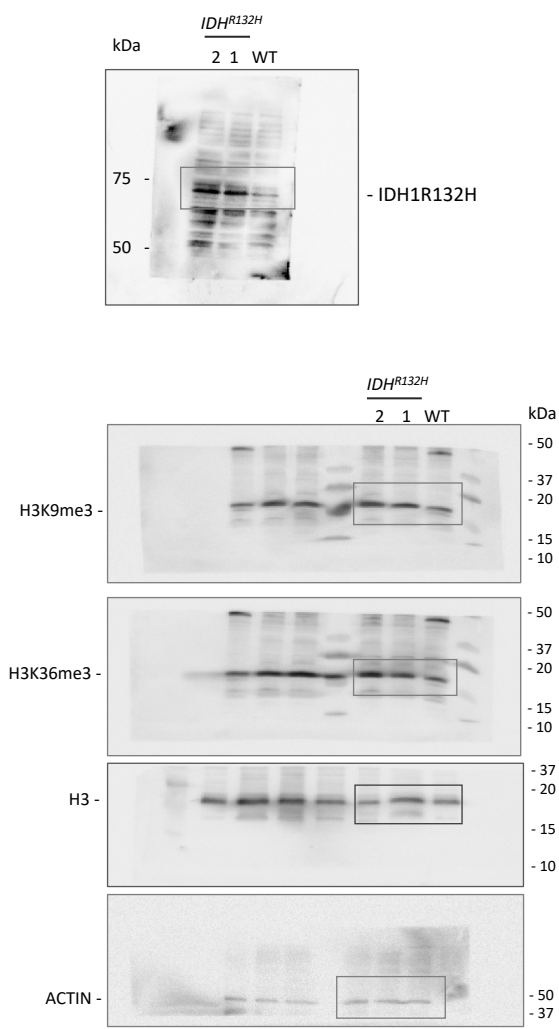

Source Data  
Figure 5



a

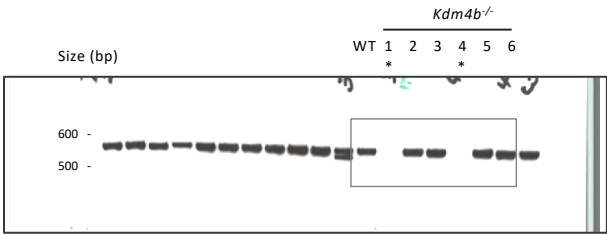

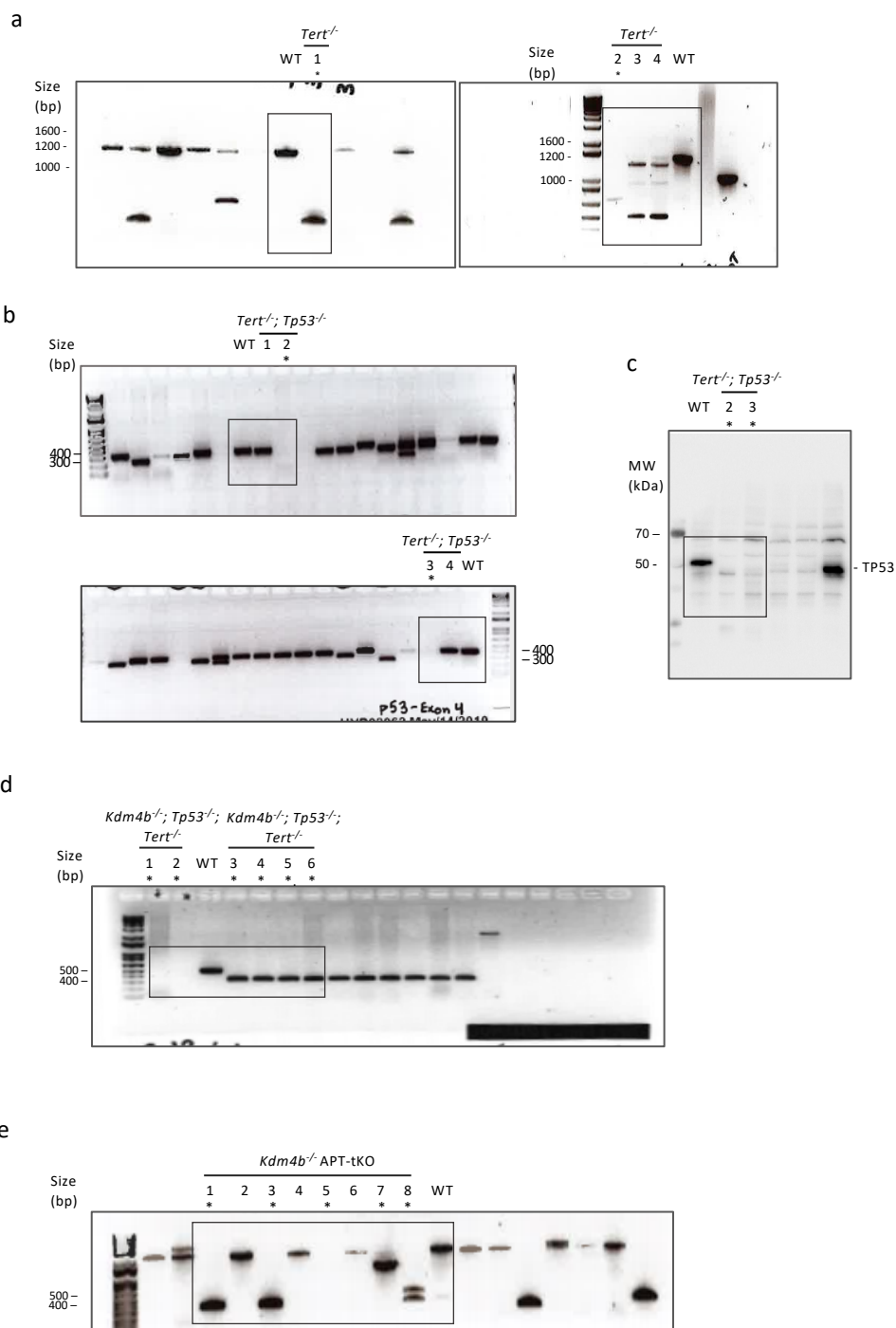

a

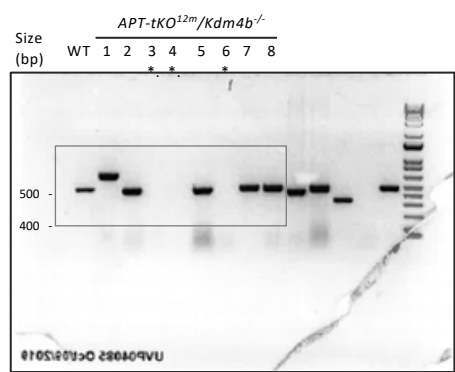

a

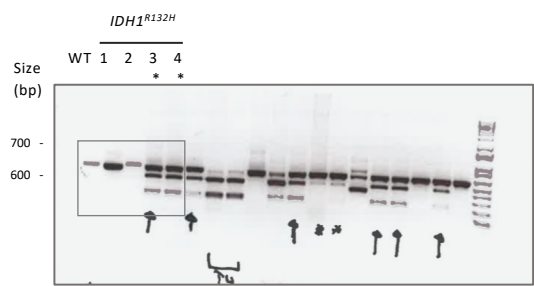

Supplement: Supplementary file 3 — Source Data [file 41467_2021_22543_MOESM3_ESM.zip › Udugama and Hii Souce data /Source Data Figures.pdf]
